# High‐Throughput Single‐Nucleus RNA Profiling of Minimal Puncture FFPE Samples Reveals Spatiotemporal Heterogeneity of Cancer

**DOI:** 10.1002/advs.202410713

**Published:** 2024-12-04

**Authors:** Weiqin Jiang, Xiang Zhang, Ziye Xu, Qing Cheng, Xiaohan Li, Yuyi Zhu, Fangru Lu, Ling Dong, Linghui Zeng, Weixiang Zhong, Yongcheng Wang, Longjiang Fan, Hongyu Chen

**Affiliations:** ^1^ Department of Colorectal Surgery the First Affiliated Hospital Zhejiang University School of Medicine Hangzhou 310003 China; ^2^ The First Clinical Medical College of Lanzhou University Lanzhou 730000 China; ^3^ Liangzhu Laboratory Zhejiang University Medical Center Hangzhou 311121 China; ^4^ Department of Laboratory Medicine the First Affiliated Hospital Zhejiang University School of Medicine Hangzhou 311121 China; ^5^ Institute of Bioinformatics and James D. Watson Institute of Genome Sciences Zhejiang University Hangzhou 310058 China; ^6^ M20 Genomics Hangzhou 310030 China; ^7^ School of Medicine Hangzhou City University Hangzhou 316021 China; ^8^ Department of Pathology First Affiliated Hospital College of Medicine Zhejiang University Hangzhou 310003 China; ^9^ College of Biomedical Engineering and Instrument Science Zhejiang University Hangzhou 310027 China

**Keywords:** FFPE sample, noncoding RNA, puncture biopsy, pseudoprogression, spatiotemporal heterogeneity

## Abstract

Puncture biopsy, especially those preserved by formalin fixed paraffin embedding (FFPE) samples, play an important role in various research purposes. Diverse single‐nucleus RNA sequencing (snRNA‐seq) techniques have been developed for FFPE samples, however, how to perform high‐throughput snRNA‐seq on small FFPE puncture samples is still a challenge. Here, the previously developed snRNA‐seq technique (snRandom‐seq) is optimized by implementing a pre‐indexing procedure for the minimal puncture FFPE samples. In analyzing 20 samples from various solid tumors, optimized snRandom‐seq still detected ≈17 000 genes and 12 000 long non‐coding RNAs (lncRNAs), achieving precise clustering based on tissue origin. A head‐to‐head comparison with 10× Genomics on fresh biopsy samples showed a similar gene detection rate, with significantly enhanced lncRNA detection, indicating that the optimized snRandom‐seq technique maintains its established gene detection advantages even when applied to small samples. Utilizing 7 puncture FFPE samples of liver metastases from 3 colorectal cancer patients pre‐ and post‐immunotherapy, the cellular developmental trajectories are reconstructed and revealed dynamic spatiotemporal heterogeneity during treatment, including insights into pseudoprogression of immunotherapy. Therefore, the optimized snRandom‐seq offers a solution for high‐throughput single‐cell RNA and non‐coding RNA analysis in minimal puncture FFPE sample.

## Graphical abstract

This study introduces an optimized single‐nucleus RNA sequencing technique (optimized snRandom‐seq) suitable for small FFPE samples, such as puncture biopsies. This method enables robust and comprehensive detection of genes and long non‐coding RNAs (lncRNAs) while revealing the dynamic spatiotemporal heterogeneity of liver metastases during immunotherapy, facilitating the inference of pseudoprogression.

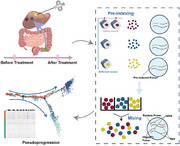

## 1 Introduction

Single‐cell RNA sequencing has provided insights into the complexity and spatiotemporal heterogeneity of the tumor microenvironment (TME),^[^ ^1^, ^2^, ^3^, ^4^ ^]^ and has paved the way for new discoveries and therapeutic strategies of cancer. Single‐nucleus RNA sequencing (snRNA‐seq), a variant of single‐cell sequencing, has proven particularly valuable for analyzing tissues that are difficult to dissociate or preserve, enabling the study of cellular heterogeneity within archived samples such as FFPE tissues. However, the complexity of single‐cell or single‐nucleus sample preparation and preservation,^[^ ^5^ ^]^ as well as the challenge of obtaining dynamic samples to reveal spatiotemporal heterogeneity^[^ ^6^ ^]^ during the treatment of patients with metastatic cancers, remain critical barriers to its broader application in basic and translational cancer research.

In the clinic, most samples, including surgical and puncture biopsy specimens, are typically formalin fixed and paraffin embedded (FFPE), which can preserve tissue structure and spatial characteristics and facilitate long‐term stable preservation.^[^ ^7^ ^]^ Percutaneous puncture sampling is an important tool in modern medicine in which tissue samples are obtained from deep inside the body of patients with inoperable tumors for diagnosis and precision medicine guidance.^[^ ^8^, ^9^, ^10^, ^11^ ^]^ Puncture specimens are a valuable resource for cancer research,^[^ ^12^ ^]^ especially for drug resistance studies in patients with metastatic tumors. Only high‐resolution analysis of spatiotemporal molecular and cellular heterogeneity in clinical specimens through dynamic repeated puncture samples can explore the mechanisms of drug resistance that emerge during treatment. Therefore, it is urgent to develop snRNA‐seq technology for small puncture FFPE samples. The development of snRNA‐seq technology for FFPE puncture samples has several technical difficulties. First, the main challenge of using FFPE samples for single‐cell sequencing is the degradation and crosslinking of RNA and DNA molecules that occur during FFPE preservation, which limits the yield and quality of single‐cell sequencing, resulting in lower sequencing efficiency and accuracy.^[^ ^13^ ^]^ Second, the low sample input from small FFPE samples obtained by biopsy poses a great challenge in obtaining sufficient amounts of cells/nuclei for high‐throughput single‐cell sequencing.

Although an increasing number of spatial transcriptomics techniques can analyze the molecular spectrum of FFPE samples, these methods lack single‐cell resolution and have limited gene detection capabilities.^[^ ^14^ ^]^ Moreover, although nuclei can be isolated from FFPE tissues, RNA crosslinking can be reversed by heating and proteinase digestion, and the popular oligo(dT)‐based RNA capture strategy is ineffective for these low‐quality samples, as demonstrated by snFFPE‐seq using the 10× Genomics single‐cell 3' solution V3 platform.^[^ ^15^ ^]^ Additionally, novel approaches have been developed for use with FFPE samples, such as snPATHO‐Seq, which employs a technique based on FFPE nuclear 10× genomics probes to capture specific gene features. However, it solely targets a small fraction of the transcriptome.^[^ ^16^ ^]^ Previously, our research team developed a droplet‐based snRNA sequencing technique, termed snRandom‐seq, for FFPE tissue by utilizing random primers to capture full‐length total RNA. In comparison to snPATHO‐seq and snFFPE‐seq, snRandom‐seq exhibits higher RNA coverage and detects a greater abundance of non‐coding RNA and nascent RNA.^[^ ^17^ ^]^ Nevertheless, snRandom‐seq necessitate a substantial amount of input material, posing challenges for the analysis of small puncture samples. Hence, to further exploit the potential of small puncture FFPE samples, there is an urgent need for the development of more applicable single‐cell sequencing methodologies.

In this study, we optimized our previously developed snRandom‐seq technique by integrating it with pre‐indexing methodologies, rendering it more applicable for minimal puncture FFPE samples. The optimized snRandom‐seq performed well in at least six common cancer types and exhibited a capacity for dynamic spatiotemporal heterogeneity analysis through repeated biopsy, providing valuable insights that aid in the identification of pseudoprogression of immunotherapy.

## 2 Results

### 2.1 Overview of the Optimized snRandom‐Seq Method for Puncture FFPE Samples

**Figure 1:**
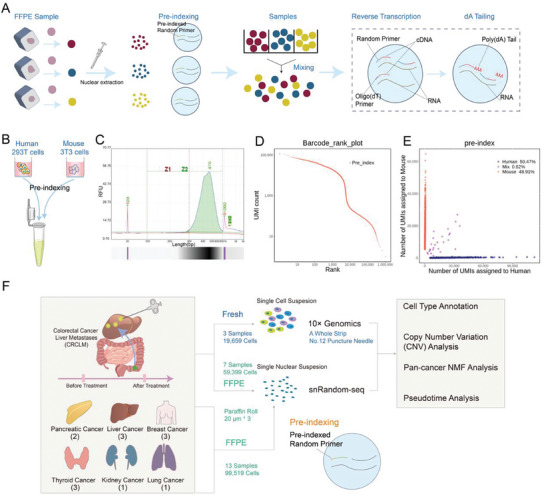
Overview of the study and the optimized snRandom‐seq method. A) Overview of the optimized snRandom‐seq method used by this study. The steps from nucleus extraction to preindexing and targeted sequencing are shown. B) Schematic diagram of experimental verification of the optimized snRandom‐seq method using a human and mouse nucleus mixture cDNA library. (C‐E) Electropherogram C), barcode plot D) and species‐mixing scatter plots E) of optimized snRandom‐seq data generated from the human‐mouse mixture library. Barcode plot for identification of the barcodes that represent true nuclei. Barcodes of the human‐mouse mixed nuclei were ordered from the largest to smallest gene counts. Species‐mixing scatter plots showing the single‐nucleus capture efficiency and doublet rate of optimized snRandom‐seq. F) A flowchart of the study. From left to right represent the samples, sequencing technologies used, and subsequent main analysis contents.

Emerging as an advanced variant of the snRandom‐seq method, the optimized snRandom‐seq protocol specializes in snRNA‐seq designed explicitly for minimal FFPE clinical samples, such as FFPE puncture samples (refer to **Figure** **1**A). This innovative adaptation is aimed at mitigating issues revolving around low cell capture rates and potential experimental fallbacks attributed to the limited count of initial nuclei in scarce FFPE samples. The process begins by isolating the region of interest from the condensed FFPE tissue block and placing it into a test tube. The tissue is then subjected to deparaffinization and rehydration with xylene and alcohol washes, which facilitate nuclear dissociation and permeabilization. To counterbalance the scarce biological tissue in puncture samples, the method strategically reduces the tissue sample size to a mere 60 µm (essentially, 3 rolls of 20 µm wax rolls). This ensures conservation of residual tissue for auxiliary tests and analyses. In accordance with the scifi‐RNA‐seq protocol,^[^ ^18^ ^]^ the optimized snRandom‐seq incorporates a pre‐indexing step during the reverse transcription phase for maximizing utility, diverging from the traditional path followed in snRandom‐seq. Here, unique pre‐indexed primers are assigned to nuclei from different samples, which are later pooled ahead of the subsequent processes. Additionally, enclosing unmodified single‐stranded DNAs securely in place is achieved by engaging multiple cycles of annealing and extending blocking primers (Figure 1A). Seeking to maximize nuclei extraction from tiny biological samples, the Dounce Homogenization method is employed for thorough cell disruption, with varying experimental parameters being fine‐tuned based on specific sample features. These parameters encompass deparaffinization time, rehydration time, lysis buffer, digestion time, homogenization iterations, lysis time, and centrifugation (refer Table S1, Supporting Information for further details). Built on prior influential work,^[^ ^19^, ^20^ ^]^ a high‐throughput single‐nucleus barcoding microfluidic platform has been developed and deployed for efficient implementation of the optimized snRandom‐seq. Upon application, high‐quality cell nuclei can be successfully harvested from a range of tumor tissues, unanimously attesting to the proficiency and adaptability of the optimized snRandom‐seq method (see Figure S1, Supporting Information for corresponding visual representation).

To confirm the accuracy and stability of the optimized snRandom‐seq technology, we still conducted a mixed‐species experiment using a blend of human and mouse cells (see Figure 1B). Significantly, the diligent use of pre‐indexing treatment was carried out on the cells before their union, subsequently leading them through the steps of single‐nucleus capture and sequencing. The created cDNA library exhibited peak fragment sizes spreading between 300 and 800 bp (visible in Figure 1C). The consistency and range of these fragment sizes removed the need for fragmentation and directly met the requirements for the application of next‐generation sequencing (NGS) techniques. The robust data analysis that followed unveiled the presence of unique and high‐quality cell nucleus barcodes. The noticeable steep slopes in the barcode‐gene rank plot (as shown in Figure 1D) provided evidence for a clear demarcation line separating true cells from background noise. Average counts revealed ≈2500 genes per cell, demonstrating the proficiency of the optimized snRandom‐seq in capturing a healthy gene expression profile per cell. Furthermore, the median ratios of mouse and human mitochondrial genes stood at ≈5% and roughly 15% respectively (for full comparison, refer to Figure S2, Supporting Information). Furthermore, the median proportions of mitochondrial genes were ≈5% for mouse cells and roughly 15% for human cells (see Figure S2, Supporting Information for a full comparison). There was no evidence of enrichment in cell populations with high mitochondrial gene proportions. Additionally, this mixing experiment demonstrated an impressively low doublet rate of just 0.62% (as illustrated in Figure 1E). These results collectively indicate that the optimized snRandom‐seq technology maintains high stability and accuracy.

To further authenticate the effectiveness of the optimized snRandom‐seq technique on small FFPE samples, we designed additional validation experiments (Figure 1F). Initially, 10 samples were obtained from pre‐ and post‐treatment biopsies of three patients who underwent immunotherapy. Among these, 7 FFPE samples were used for temporal heterogeneity analysis. Additionally, three FFPE samples and three fresh samples were used to compare the performance of optimized snRandom‐seq with the currently mainstream 10× Genomics technology. Subsequently, we executed a series of optimized snRandom‐seq experiments on samples collected from six different common cancer types (Figure 1F). Leveraging the data generated by optimized snRandom‐seq, further data analyses were conducted, including cell type annotation, copy number variations (CNV), pan‐cancer analysis, and pseudotime analysis.

### 2.2 Comparison of the Optimized snRandom‐Seq with 10× Genomics

**Figure 2:**
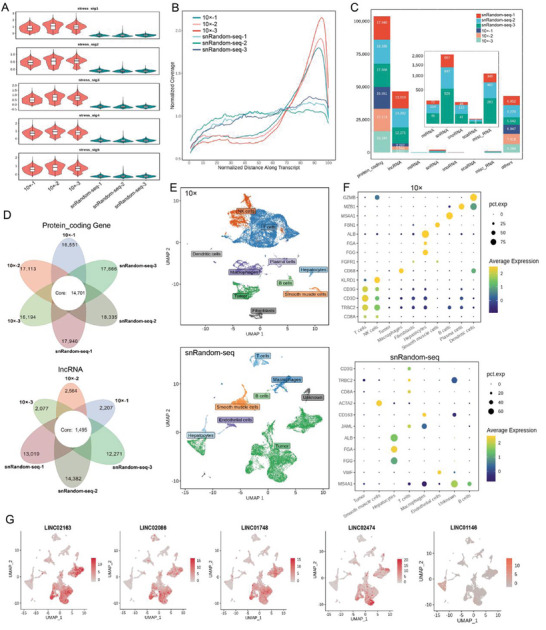
Comparison of the optimized snRandom‐seq and 10× Genomics technologies based on paired‐sample analysis. A) Violin plots and boxplots of relative expression of five artefactual stress‐associated gene expression signatures. B) Read distribution along the transcript body in different samples. C) Counts of different RNA biotypes detected in each sample. D) A petal diagram was used to illustrate data for protein‐coding genes and long noncoding RNAs detected by 10× Genomics and optimized snRandom‐seq. The shared number of features between samples is represented in the center, while the total number of features detected in each sample is indicated at the edges. E) UMAP visualization of integrated clustering results of FFPE and fresh biopsy samples, with cell type annotation information. F) A dot plot is used to illustrate annotation of marker genes in different cell types. G) Identification of cancer cell‐specific expression of lncRNAs using optimized snRandom‐seq.

To verify whether the optimized snRandom‐seq technique maintains its established gene detection advantages in small‐sample analysis, we conducted a direct comparison using three FFPE samples matched with fresh samples analyzed by the widely used 10× Genomics technology. Due to the enzymatic dissociation method used in the preparation of the cell suspension by 10× Genomics, a high degree of processing‐related artefacts was observed, as expected, in the RNA data generated by 10× Genomics, including the stress response pathway (**Figure** **2**A; Table S2, Supporting Information). In contrast, lower expression of stress‐related genes was observed in the data generated by the optimized snRandom‐seq. These stress signals may introduce bias in the interpretation of these pathways in scRNA data. As expected, the optimized snRandom‐seq data exhibited more uniform coverage across an entire transcript, while 10× Genomics showed a peak in single‐end reads at the 3’ end (Figure 2B). Based on the total number of reads detected per cell and the corresponding number of features, the detection efficiency of optimized snRandom‐seq was comparable to that of 10× Genomics (Figure S3A, Supporting Information). Based on the ratio of spliced and unspliced reads, the proportion of unspliced reads in optimized snRandom‐seq data was nearly three times that of genomic technologies, partially because FFPE samples can only be analyzed by nuclear extraction (Figure S3B, Supporting Information). The total number of genes detected in FFPE small samples was similar to that in fresh samples, both at ≈17 000, with a high overlap proportion (Figure 2C,D). Additionally, both techniques detected a number of lncRNAs, consistent with the presence of a polyA structure for some lncRNAs. Due to the random primer characteristics of optimized snRandom‐seq, the number of lncRNAs identified was nearly six times higher than the 2000 detected by 10× Genomics, reaching over 12 000 (Figure 2C,D). In addition, the optimized snRandom‐seq detected many short noncoding RNAs, including small nucleolar RNAs (snoRNAs), small nuclear RNAs (snRNAs), and microRNAs (miRNAs) (Figure 2C). In terms of the coverage of transcript regions, 10× Genomics focused more on exonic and UTR regions, whereas the optimized snRandom‐seq covered more intronic regions (Figure S3B, Supporting Information). These findings collectively indicate that the optimized snRandom‐seq technique maintains its previously (snRandom‐seq) demonstrated gene detection advantages when analyzing small sample.

We further performed integrated analysis of two pairs of head‐to‐head data and tumor cell annotation based on copy number variation (CNV). Similar to 10× Genomics, the tumor cells identified by the optimized snRandom‐seq showed significant CNV differences on chromosomes compared to normal cells. In this study, we directly labeled tumor cells based on CNV analysis (Figures S4–S6, Supporting Information). Annotation of cell types and their coverage by the two sequencing technologies showed good consistency. Although 10× Genomics identified more cell types, such as dendritic cells and plasma cells, their proportion was relatively small and could be attributed to the difference in the starting sample amount (Figure 2E).

While both technologies identified a consistent number of cell types, their proportions differed significantly. The 10× Genomics method captured more immune cells, whereas the optimized snRandom‐seq identified more cancer cells (Figure 2E; Figure S3C, Supporting Information). This discrepancy led to differences in integration effects: 10× Genomics achieved better integration by primarily capturing normal immune cells, while optimized snRandom‐seq's integration was less effective due to the high heterogeneity of cancer cells, which may also be attributed to differences between single‐cell and single‐nucleus assays (Details are described in the Discussion section). Nevertheless, optimized snRandom‐seq effectively integrated normal cells such as T cells and endothelial cells, indicating no sample bias in cell capture (Figure S7, Supporting Information).

Marker gene expression dot plots validated the annotation accuracy of both technologies (Figure 2F). Differential gene expression analysis revealed highly specific DEGs with minimal cross‐contamination (Figure S8, Supporting Information), and GO enrichment analysis confirmed cell‐type‐specific pathways, such as B cell receptor signaling in B cells, with a high overlap in enrichment results (Figure S9, Supporting Information), supporting the reliability of optimized snRandom‐seq.

Interestingly, the optimized snRandom‐seq still identified several lncRNAs specifically expressed in cancer cells and associated with cancer development and progression (Figure 2G; Tables S3 and S4, Supporting Information). While lncRNAs play critical roles in cancer, their expression in the tumor immune microenvironment (TIME) is largely unexplored due to their cell type‐specific nature and the lack of specific annotations.^[^ ^21^ ^]^ Our results provide new insights into clinically relevant lncRNAs in malignant cells within the TIME.

### 2.3 Performance of the Optimized snRandom‐Seq in FFPE Puncture Samples of Six Common Cancer Types

#### 2.3.1 Overall Performance

**Figure 3:**
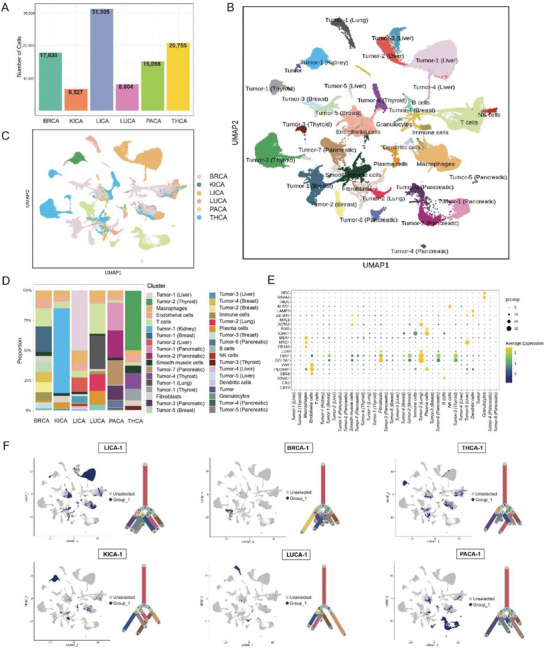
Performance of the optimized snRandom‐seq in FFPE puncture samples of six cancer types. Samples of liver cancer, lung cancer, kidney cancer, breast cancer, pancreatic cancer, and thyroid cancer were used. A) The number of single cells detected across the six types of cancer. B) UMAP plot of the integrated results of the 13 samples across six types of cancer. C) UMAP plot of the integration status of the different types of cancer, highlighting similarities and differences between clusters. D) The bar graph displays the proportional distribution of cell clusters across different types of cancer. E) Incorporate dot plots to depict the expression profiles of marker genes across different cell types. F) The UMAP plot displays the location of nauseating tumor cells identified in the above clustering results, with the corresponding evolutionary tree of tumor cells based on CNV information plotted on the right side.

To further validate applicability of the optimized snRandom‐seq technology to multiple tumor tissues, we selected FFPE samples from six types of cancer for the optimized snRandom‐seq (Figure 1F; details of the sample information can be found in Table S5, Supporting Information). These six cancer types included lung cancer (LUCA, 1 sample), liver cancer (LICA, 3 samples), kidney cancer (KICA, 1 sample), thyroid cancer (THCA, 3 samples), pancreatic cancer (PACA, 2 samples), and breast cancer (BRCA, 3 samples) (Figure 1F). After quality control, 17,830 cells for BRCA, 6,527 cells for KICA, 31,385 cells for LICA, 8004 cells for LUCA, 15 098 cells for PACA, and 20 755 cells for THCA were assessed by the optimized snRandom‐seq (**Figure** **3**A). Similar to previous samples, the median UMI detection for the 13 samples was ≈1500, and the median gene detection was 1000‐2000. Mitochondrial content was low (< 3%) in most samples, indicating high data quality (Figure S10A, Supporting Information). All 13 samples showed a median of over 80% unspliced transcripts, consistent with prior results, due to our focus on nuclear RNA and the use of random primer amplification for even transcript coverage (Figure S10B,E, Supporting Information). Most reads mapped to exonic regions, with some to intergenic and UTR regions (Figure S10C, Supporting Information). The detection of substantial lncRNA and a consistent number of protein‐coding genes across samples confirm the reliability of the optimized snRandom‐seq (Figure S10D, Supporting Information).

To facilitate subsequent annotation integration, we first performed CNV analysis and tumor cell identification for the 13 cancer samples. The results showed that the data of each sample generated by the optimized snRandom‐seq can be used to clearly distinguish tumor cells from normal cells, with tumor cells exhibiting significant copy number variation information (Figure S11, Supporting Information). Furthermore, we conducted an integrated analysis of the 13 sample datasets; the final clustering results are shown in Figure 3B. Many specific cell populations in different cancer types were identified (Figures S12 and S13, Supporting Information); we also detected a certain number of integrated and rational populations, such as Cluster #2 and Cluster #3, which exhibited good homogeneity and were present in a certain proportion in different cancer types (Figure 3C,D; Figures S12 and S13, Supporting Information). Based on marker genes (Figure 3E), cells were annotated as macrophages and endothelial cells. The specific populations were mainly composed of tumor cells identified through CNV analysis in the integrated clustering results (Figure 3F; Figure S14, Supporting Information). For example, Cluster #0 was composed of tumor cells identified in samples LICA‐1 and LICA‐3; the tumor cells of sample LICA‐2 were grouped into Clusters #7 and #19. The results potentially indicate the heterogeneity of tumor cells among different samples from the same cancer type.

#### 2.3.2 Cellular lncRNA Heterogeneity Within the Six Common Cancers

**Figure 4:**
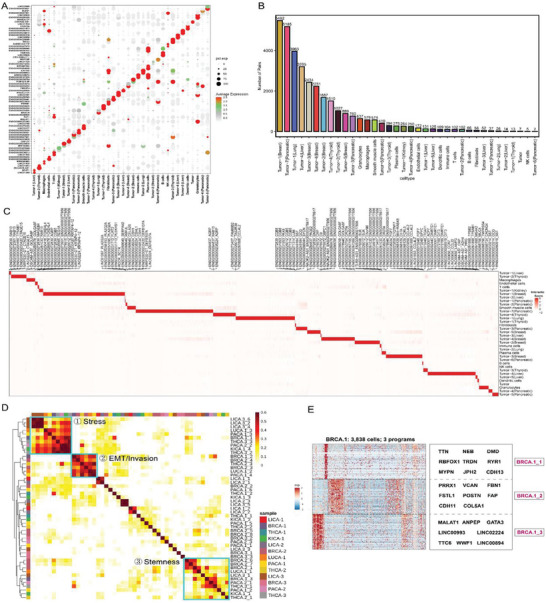
Pancancer analysis of optimized snRandom‐seq data for 13 cancer samples of six common cancers. A) A dot plot was used to show expression of the top 2 ranked lncRNAs among the different clusters, as identified as differentially expressed genes. B) The LncPairs algorithm operates to pinpoint lncRNA‐mRNA pairs that are specific to each cell type, quantifying the distinct pairs within each. C) A heatmap is utilized to visually compare and display the cell‐specific lncRNA‐mRNA pairs across various cell types, with a special emphasis on denoting the three most prominent lncRNA‐mRNA pairs. D) Pairwise similarities between nonnegative matrix factorization (NMF) programmes identified across all the cancer samples analyzed and ordered by hierarchical clustering. Three clusters or recurrent heterogeneous programmes (RHPs) are indicated by squares and numbers. E) A heatmap shows relative expression of genes from three programmes across all cells in breast cancer sample 1 ordered by hierarchical clustering. NMF programmes are annotated (right), and some selected genes are indicated (left).

We focused on identifying differentially expressed lncRNAs in addition to a large number of differentially expressed protein‐coding genes. **Figure** **4**A shows a dot plot of the top two differentially expressed lncRNAs identified for each cluster. The diagonal pattern in the graph clearly indicated the expression specificity of lncRNAs in the cluster. In addition, some lncRNAs have been previously reported,^[^ ^22^, ^23^ ^]^ such as LINC00958 in liver hepatocellular carcinoma (HCC), which sponges miR3619‐5p to upregulate expression of liver cancer‐derived growth factor (HDGF), promoting HCC progression and adipogenesis, and directly acts on NUDT19 to activate the mTORC1/P70S6K signaling pathway. Overexpression of NUDT19 and the mTORC1 activator MYH1485 reverse the inhibitory effects of LINC00958 silencing on HCC proliferation, migration, and epithelial‐mesenchymal transition (EMT),^[^ ^22^, ^23^ ^]^ and a large number of newly identified lncRNAs that are specifically expressed in tumor groups were found (Figure 4A; Table S6, Supporting Information). To further confirm the cell‐specific lncRNAs’ function, we adopted LncPairs algorithm for lncRNA‐mRNA interactions annotation.^[^ ^24^ ^]^ As noted in Figure 4B, our study uncovered many lncRNA‐mRNA pairs, e.g. 5482 pairs specific to tumor clusters in breast cancer – the top number to other cell types and cancer forms. Within the immune microenvironment, granulocytes and macrophages presented high numbers of these pairs, specifically counting 637 and 579 respectively. Interpreting from the heat map, it is evident that distinct cell clusters express unique lncRNA‐mRNA pairs, displaying a striking cluster specificity further illustrated and explored in Figure 4C. The LncPairs results independently validated the functionality of identified lncRNAs in lncRNA‐mRNA interactions and cancer regulations. These lncRNAs can serve as the basis for future research and provide a deeper understanding of the differences in cell types and molecular mechanisms among different cancer types.

#### 2.3.3 Recurring Expression Programs of Common Cancers

Pan‐cancer analysis can provide an accurate understanding of heterogeneity and commonality across different cancer types. To explore the existence of recurring expression programmes among six types of cancer, we performed nonnegative matrix factorization (NMF) on tumor cells identified from the 13 samples. We repeated the NMF analysis with distinct parameters to identify robust expression programs, each defined by the top 50 genes based on NMF scores (Table S7, Supporting Information). Overall, we detected 57 robust expression programmes across all cancer types and 3–8 programmes in individual samples. The NMF programmes were subjected to hierarchical clustering based on their shared genes, which highlighted the presence of multiple recurrent heterogeneous programs (RHPs) of gene expression across multiple samples (Figure 4D). Although we used a limited number of only 13 samples, ultimately, we identified three RHPs (stress, EMT/invasion, and stemness) based on functional enrichment analysis of the top 50 genes representing each RHP (Figure 4D; Figure S15, Supporting Information). Stress and stemness programmes were observed in all six cancer types. The EMT/invasion programme was only present in four cancer types, including lung cancer, breast cancer, pancreatic cancer, and thyroid cancer. However, compared to the stress programme and EMT/invasion programme, the stemness programme showed greater variability across different cancer types. This suggests a certain degree of similarity in the stemness activity of tumor cells among different cancer types, with the similarity being lower than their EMT/invasion or stress response characteristics.

A heatmap clearly shows distinct cell populations in the sample, with tumor stem‐cell‐specific features and strong invasive ability caused by the epithelial‐mesenchymal transition (Figure 4E). Moreover, we found some known stemness‐related genes among the top 50 genes representing the stemness programme, such as the transcription factor GATA3, which has been identified as a robust predictor of clinical outcome in human luminal breast cancer. Within the mammary gland, GATA3 plays a critical role in the differentiation and commitment of luminal epithelial cells.^[^ ^25^ ^]^ Interestingly, we also identified several lncRNAs among the top 50 genes representing the stemness programme, which may serve as a basis for investigating the role of lncRNAs in breast cancer stemness.

Taken together, we found that the data obtained from the optimized snRandom‐seq processing of small samples of different cancers retains the characteristics of the cancer itself. After pancancer NMF analysis, the main features of cancer, such as stemness and EMT/invasion, can be obtained, and genes or even lncRNAs that are commonly or specifically associated with them in different types of cancers can be identified.

### 2.4 Identification of Pseudo‐Progression of Immunotherapy Based on Dynamic Spatiotemporal Heterogeneity

**Figure 5:**
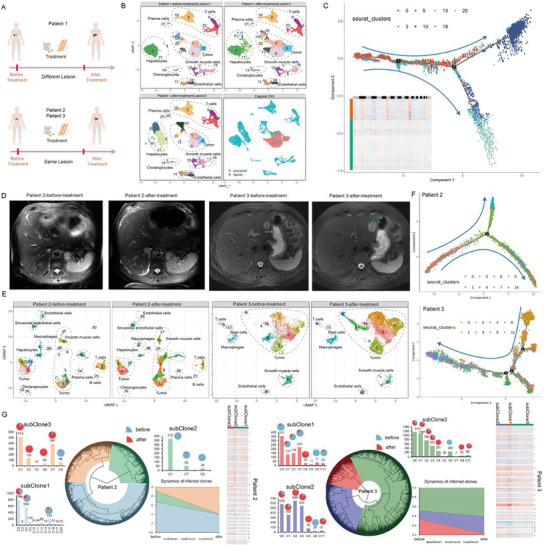
Temporal heterogeneity of tumor cells in pre‐ and post‐treatment FFPE biopsy samples from two patients. A) Schematic diagram of the experimental design, where three samples from Patient 1 were derived from two different lesions, while four samples from Patients 2 and 3 were derived from the same lesion (two sample from each patient), respectively. B) The UMAP plots display the clustering results for the three puncture samples for Patient 1, with the first three UMAP plots corresponding to these samples. The UMAP plot in the lower right corner shows the CopyKat CNV identification results for tumor cells. C) The UMAP plot illustrates the developmental trajectory of tumor cells of Patient 1, with the heatmap in the lower left showing CNV variation in cells; darker colors indicate greater CNV variation. D) Imaging results from the same lesion before and after treatment in two patients. E) UMAP plots were generated for patients before and after treatment, highlighting the clustering and annotation of samples from two patients and the similarities and differences between clusters after treatment. For example, Clusters #2 and #6 were only present in Patient 1's samples after treatment; Cluster #4 was only present in Patient 2's samples after treatment. F) We performed pseudotime analysis on tumor cells to demonstrate their differentiation trajectory in the two patients. G) The circular phylogenetic tree displays the lineage and subclonal information of tumor cells. The adjacent bar graph shows the proportion of cell cluster composition corresponding to each subclone. Simultaneously, the stacked plot in the lower right corner illustrates the trend in the proportion of tumor cell subclones before and after treatment, as determined by serial biopsies. The heatmap displays the copy number variations of each tumor subclone. Red represents gain, blue represents loss.

Here, we selected seven core needle FFPE specimens both pre and post immunotherapy, from the identical lesion in three colorectal liver metastasis (CLM) patients (**Figure** **5**A). In the seven samples, three samples from Patient 1 were obtained from two different lesions, while four samples from Patient 2 and Patient 3 (two samples obtained from a same lesion of each patient), respectively. After treatment, Patient 1 showed effective response with tumor shrinkage on imaging. Patients 2 and 3 were assessed as having progressive disease (PD) according to RECIST criteria, with substantial tumor enlargement on imaging (Figure 5D). Patient 3 decided to continue immunotherapy because the tumor marker carcinoembryonic antigen (CEA) was on a downward trend and subsequently experienced partial response (PR). We inferred developmental trajectories of these tumors at the single‐cell level pre‐ and post‐treatment, and identified the pseudoprogression of immunotherapy in one of the patients, accordingly.

From the UMAP plot in Figure 5B, it is evident that the three samples from Patient 1, aside from hepatocytes and tumor cells, show considerable consistency. The CNV results by Copykat further validate the accuracy of tumor cell identification, with greater CNV variation observed in Clusters 0, 3, 5, and 13. Interestingly, Cluster 10 is specific to Lesion 2, while Cluster 18 is unique to Lesion 1, indicating that tumors from different lesions exhibited some heterogeneity. Using pseudotime analysis (Figure 5C), we reconstructed the developmental trajectories of tumor cells in Patient 1 before and after treatment, showing progression from higher malignancy (Clusters 0, 3, 5, 13) to lower malignancy (Clusters 10, 18, 20). Notably, the two lesions displayed different developmental trajectories. The results not only confirm the image‐based efficacy evaluation of Patient 1 from a single‐cell genomics perspective, but also underscore the reliability of the optimized snRandom‐seq technology.

For Patients 2 and 3, where lesions were enlarging, we conducted similar and more detailed analyses. Based on annotation results, we identified distinct cell populations, including endothelial cells, T cells, and macrophages, among others (Figure 5E). Subsequently, using the CNV score for tumor cell detection, we classified clusters #0, 1, 2, 4, 6, 7, 9, and 14 as neoplastic cells in Patient 2, and clusters #0, 1, 2, 3, 4, 6, 8, and 11 as tumor cells in Patient 3 (Figure 5E; Figure S16A,B, Supporting Information). Intriguingly, there were major changes in the composition of tumor cells in both patients before and after treatment. Certain tumor cell clusters were specifically present in the post‐treatment samples, suggesting a potential transformation during immunotherapy (Figure 5E). For instance, in Patient 2, we found that clusters #2 and #6 were exclusive in post‐treatment samples, whereas, in Patient 3, clusters #4 and #8 were specifically found in post‐treatment samples, and cluster #6 was present in pre‐treatment samples. We also conducted DEG analysis for identified clusters (Tables S8 and S9, Supporting Information) and performed enrichment analysis using MSigDB which efficiently depict intrinsic and extrinsic cancer pathogenesis, encompassing cellular signaling, inflammation, proliferation, and metabolism.^[^ ^26^ ^]^ Based on the heatmap result, malignant cell clusters, particularly Cluster #0, 1, 4, 7, and 14 in Patient 2, and Cluster #0, 1, 2, 3, and 11 in Patient 3, were notably enriched in hallmark gene sets. Interestingly, some clusters, like Cluster #2 and 6 in Patient 2 and Cluster #8 in Patient 3, exhibited opposing enrichment posttreatment (Figure S17, Supporting Information), suggesting potential drug response phenomena.

To further explore the changes in malignant cells during treatment, we performed pseudotime analysis on the identified malignant cells in Patient 2 and Patient 3, visualizing their developmental trajectories (Figure 5F; Figure S18, Supporting Information). In Patient 2, malignant cells exhibited prominent branching, with clusters #0, 4, and 14 at the trajectory start point. One branch progressed toward clusters #1, 7, and 9, another towards cluster #6, which almost exclusively emerged post‐treatment, suggesting novel tumor cell alterations. Further characterizing tumor cell features, we divided the tumor cells into three tumor subclones using the CNV matrix (Figure 5G). Overall, post‐treatment, subClone2 disappeared, and subClone3 proportion increased (Figure 5G). Interestingly, subClone1 comprised post‐treatment Cluster #0 cells and pre‐treatment Clusters #4 and 14, primarily positioned at the early trajectory stages, while subClone2 mainly contained pre‐treatment Clusters #1, 7, and 9. SubClone3 consisted of post‐treatment Clusters #1, 2, 6, indicating that subClone2 and 3 occupied the developmental trajectory's two branches, representing two developmental directions of tumor cells. From the CNV heatmap, subClone2 and 3 exhibited clearer CNV than subClone1 (Figure 5G), revealing the continued malicious progression of tumor cells in Patient 2 post‐treatment. Similarly, we conducted the same analysis for Patient 3. Unlike Patient 2, only minor branches were observed in Patient 3 (Figure 5F), with most malignant cells primarily clustering along the dominant developmental trajectory, culminating in clusters #4 and #8 (Figure S18, Supporting Information). Based on prior hallmark gene‐set enrichment analyses, Cluster #8 showed no enrichment of any cancer‐related features (Figure S17, Supporting Information), indicating the malignant cell subgroups in Patient 3 responded to treatment, progressing towards benignancy. Furthermore, to validate our identification results, we conducted FISH verification on defined marker genes of the most significantly altered cluster #4 in Patient 3 (Table S9, Supporting Information), shown in Figure S19 (Supporting Information). Post‐treatment samples displayed significantly increased expression in multiple regions. Concurrently, through subclonal analysis, subClone1 disappeared post‐treatment, indicating that almost all malignant tumor cell clusters responded to therapy (Figure 5G). In comparison to subClone1, the new subclones found post‐treatment, subClone2 and SubClone3, displayed fewer CNVs, especially subClone3 (Figure 5G). This reveals that while the tumor lesion of Patient 3 was enlarged on imaging, but in fact, the tumor cells were responsive to treatment and progressing towards benignancy, which is a pseudoprogressive phenomenon of immunotherapy.

Through trajectory construction, we were also able to obtain potential molecular mechanisms of tumor cells during development, such as identification of development‐related genes (Figure S20, Tables S10 and S11, Supporting Information). This gene information will provide a reference for clinical treatment. Similar to previous findings, capture of lncRNAs by the optimized snRandom‐seq will also provide more information on lncRNAs related to tumor development. In conclusion, the results demonstrate that the optimized snRandom‐seq can be used in time‐series analysis of tumor cells.

## 3 Discussion

In this study, to address the challenge of low sample quantity in minimal puncture FFPE specimens, we upgraded the previously developed snRandom‐seq, which offers several advantages over widely used 10× genomics technology. First, by utilizing pre‐indexing to label multiple samples and multiplexed detection, we addressed the low input issue of single puncture sample. Second, by incorporating the use of random primers,^[^ ^17^, ^18^, ^19^, ^20^ ^]^ we comprehensively and sensitively captured total RNA from a single nuclei, overcoming the technical difficulty of achieving single‐nucleus sequencing of FFPE samples due to RNA fragmentation.

To our knowledge, this is the first study to compare the optimized snRandom‐seq and traditional 10x Genomics, based on three puncture FFPE matched with fresh samples. Our study demonstrated that the optimized snRandom‐seq also has advantages in capturing noncoding RNAs, such as lncRNAs and miRNAs. Many of the identified lncRNAs in the manuscript have been validated in prior research studies. For instance, the research conducted by Ma et al.^[^ ^27^ ^]^ elucidates the negative correlation between the overexpression of LINC02163 in CRC tissues/cell lines and patient prognosis. Concurrently, it reveals that attenuating LINC02163 expression dramatically reduces CRC cells' proliferation and metastasis. In a similar vein, Tian et al.^[^ ^28^ ^]^ observed elevated LINC02418 levels in human cancer specimens compared to surrounding healthy tissues. They associated this high expression with poor patient prognosis, suggesting that LINC02418 enhances CRC progression by augmenting tumor growth and cellular movements. Counterbalancing these oncogenic lncRNAs are tumor‐suppressing lncRNAs, such as LINC01146, which has shown a unique expression pattern. Overexpression of LINC01146 has shown to inhibit tumor growth, whereas its reduction encourages tumor growth.^[^ ^29^ ^]^ Different cancers, like lung, liver, and breast cancers, have shown a connection with either the promotion or inhibition of lncRNAs.^[^ ^30^, ^31^, ^32^ ^]^ Owing to this, lncRNAs have been identified as potential cancer biomarkers.^[^ ^33^ ^]^ The expression and variability of lncRNAs at the single‐cell level, however, remain unclear. The development of a new database (http://rna.sysu.edu.cn/colorcells/) addressing this issue by cataloging specific lncRNAs based on single‐cell RNA data is hence a remarkable leap.^[^ ^34^ ^]^ The recent advent of the optimized snRandom‐seq's ability to account for lncRNAs abundance will further deepen our understanding and potentially develop novel noncoding RNA strategies to improve cancer treatment response.

Existing research has indicated a proportional discrepancy between single‐nucleus and single‐cell RNA sequencing in cellular composition, particularly showing a relative depletion in immune cells and an increased proportion of tumor or substantive cell.^[^ ^35^, ^36^ ^]^ Consistent with previous results, we captured a higher proportion of tumor cells and a smaller proportion of immune cells through nuclear extraction, in contrast to the fresh tissues needed for 10× Genomics. Compared to the proportion of tumor cells determined based on morphological observation through staining, it appears that nuclear extraction may better reflect the true cellular composition of the tissue. It has been well documented that different cell types exhibit different dissociation efficiencies during the dissociation process, with fibroblasts and endothelial cells usually being more embedded in the extracellular matrix and basement membrane, making them more difficult to dissociate.^[^ ^37^ ^]^ At the same time, some sensitive cells may be fragmented due to excessive dissociation, likely resulting in the inability to effectively capture all cell types of a tissue during the dissociation process, which will greatly affect the accuracy of results. However, this is still an open question and needs more evidence in the future.

Due to the characteristics of the puncture technique itself, the sample volume obtained is often very small, and sampling error and individual variations can lead to differences in the cellular composition of samples from the same tissue,^[^ ^38^ ^]^ as shown in our results for samples from three different CLM patients. Compared to Patient 3, more liver hepatocyte cells and immune cells were captured for Patient 2, and the quantity of immune cells was negligible to the point of being inconsequential. However, through the optimized snRandom‐seq data, we were able to capture a large number of tumor cells in samples from multiple types of cancer, which can be used for studies on tumor heterogeneity, drug sensitivity or resistance, as well as other research areas, and provide assistance in formulating treatment plans. Despite the natural shortcoming of sampling error with puncture samples, analyzing the temporal heterogeneity of dynamic repeated puncture of the same sample and the spatial heterogeneity of different lesions at the same time point is still the best strategy for studying acquired drug resistance during treatment of patients with advanced tumors. The developmental trajectories of tumor cells in the three CLM patients during treatment and the pseudotime analysis results were consistent with the clinical outcome, which was helpful to identify the pseudo‐progression on imaging. As we know, the “pseudo‐progression” phenomenon poses a huge challenge for immunotherapy, as tumors may seem to expand and clinicians have misconceptions about the efficacy, leading to a failure of subsequent treatment decisions. Therefore, it is expected to provide personalized precision medicine guided by the analysis of tumor biological heterogeneity, and make up for the shortcomings of traditional imaging in judging the pseudo‐progress of immunotherapy.

There were some limitations in our research. Notably, the number of samples in our pan‐cancer and pseudotime analysis was limited. We mainly captured obvious features between different cancer types, such as stemness and EMT. Meanwhiles, in our pseudotime analysis, the more samples different lesions and time points are used, the better we can identify the dynamic changes that the tumor presents at the molecular level during treatment. It is expected that the aforementioned limitations will be addressed in our next investigation in the future, and the snRandom‐seq will play a great role in the study of the spatiotemporal heterogeneity of tumor.

## 4 Experimental Section

### Experimental Model

293T cells and 3T3 cells were obtained from Procell Life Science & Technology. The collection and study of human samples in this research was approved by the Research Ethics Committee of the First Affiliated Hospital, Medical School of Zhejiang University (Approval No.: IIT20220893A). Prior to the initiation of this study, informed written consent was obtained from all participants or their immediate relatives. All FFPE biopsy samples of clinical human cancers were provided by the First Affiliated Hospital, Medical School of Zhejiang University. The clinical details of the samples can be found in Table S5 (Supporting Information).

### Species Mixture Experiment

HEK293T cells and 3T3 cells were cultured in Dulbecco's modified Eagle's medium (DMEM) supplemented with 10% (v/v) heat‐inactivated foetal bovine serum (FBS) at 37°C in a 5% CO2 incubator and passaged every two days. For the species mixture experiment, the cells were harvested and washed three times with phosphate‐buffered saline (PBS) by centrifugation at 4 °C and 600 × g for 3 min. The cells were lysed using precooled nuclei lysis buffer (1X PBS with 0.1% Nonidet P‐40 (NP‐40) and 1 U µL^−1^ RNase Inhibitor) by incubating at 4 °C for 5 min. The fresh nuclei were then washed three times and fixed by adding 1 mL of 4% paraformaldehyde (PFA) to PBS and incubating at room temperature for 15 min. The PFA was then removed by centrifugation at 600 × g for 3 min, and the nuclei were washed three times with 1 mL of precooled wash buffer (1X PBS with 1 U µL^−1^ RNase Inhibitor). The nuclei were permeabilized by adding 500 µL of 0.1% Triton X‐100 diluted in precooled wash buffer and incubated at 4 °C for 5 min. Then, 1 mL of wash buffer was added directly to the nuclei, which were washed three times with 1 mL of precooled wash buffer. The HEK293T nuclei and 3T3 nuclei were counted and mixed equally. The mixture was then processed for single‐nucleus RNA‐seq according to the following snRandom‐seq protocol.

### Isolation of Tissue Cells

Human liver cancer tissue samples were dissected and minced and then placed in digestion media consisting of Liberase DL (400 µg mL^−1^) and elastase (100 µg mL^−1^) in RPMI (Gibco 72400120). The samples were partially dissociated using a gentleMACS Dissociator, followed by a 30‐minute incubation in a Nutator at 37 °C, and then dispersed to a single‐cell suspension. The samples were then treated with processing buffer (5% foetal bovine serum in PBS) and DNAse I (100 µg mL^−1^) and incubated at 37 °C for 5 min before being cooled to 4 °C for the remainder of the protocol. The cells were filtered through a 100 µm filter and pelleted. The buffer was inactivated by adding excess processing buffer, and the cells were filtered through a 70 µm strainer and pelleted again before being resuspended.

### scRNA‐Seq Library Construction

Approximately 18 000 cells were loaded onto a Single Cell A Chip and processed using Chromium Controller and Chromium Single Cell 3.0 Reagent Kits v2 (10× Genomics, Pleasanton, CA) to generate single‐cell GEMs (gel beads in emulsion). The resulting scRNA‐seq libraries were prepared using Chromium Single Cell 3.0 Gel Bead and Library Kit (P/N #120236, 120237, 120262; 10x Genomics). The DNA library was qualitatively analyzed with an Agilent 2100 Bioanalyzer, and the concentration was measured by a Qubit (Invitrogen). The libraries were sequenced using an Illumina NovaSeq sequencer (Genergy Biotechnology Shanghai). The raw scRNA‐seq dataset consisted of Read1, Read2, and i7 index reads, with the 16 bp 10x barcode and 10 bp UMI sequence contained in 26‐bp Read1; 98‐bp Read2 contained the sequence of the cDNA fragment.

### Single Nucleus Isolation from FFPE Puncture Samples

FFPE samples were cut from paraffin blocks with a size of 20 µm per section and 3 sections per sample and then washed twice with 1 mL of xylene at room temperature for 5 min each to remove the paraffin. Considering the low starting material of some samples, we reduced the number of washes to 1 for specific samples. As shown in Table S1 (Supporting Information), we performed only one wash for lung cancer, pancreatic, and thyroid samples. The samples were then gently dehydrated by immersing them in a series of graded ethanol solutions (from 100% pure ethanol to 30% ethanol). The samples were washed twice with precooled wash buffer (125 µm glycine, 2 mM MgSO4 in 3X SSC buffer) and homogenized with a Dounce homogenizer on ice in the presence of precooled lysis buffer, as shown in Table S1 (Supporting Information). We used different lysis buffers and different lysis times for different samples. The homogenization times were also adjusted according to the sample type. After homogenization, the Doune homogenizer was washed with 1 mL lysis buffer, and 100 µL of 10 mg mL^−1^ protease K was added to the lysis buffer and incubated at 37 °C for 5 min. The separated nuclei were filtered through a 20 µm cell strainer and washed twice with wash buffer. Equal portions of nuclei were stained with DAPI (4',6‐diamidino‐2‐phenylindole), loaded into a blood cell counter, and observed under an inverted fluorescence microscope.

### snRandom‐Seq Library Preparation

To achieve sufficient numbers of cells, a preindexing step was integrated into the reverse transcription step based on published scifi‐RNA‐seq protocols. The extracted nuclei were labelled with barcoded cDNA using preindexing random primers and then merged prior to subsequent steps. Then, qualified single nuclei were subjected to single‐nucleus RNA‐seq processing according to a previously published snRandom‐seq protocol.^[^ ^17^ ^]^ Detailed procedures, including the volumes of lysis buffer and permeabilization buffer, reaction system, and reaction programme, are provided in the supplementary materials of the previous publication.

### Data Analysis

*Preprocessing of* 10× *Genomics Data*: Sequences from NovaSeq analysis were demultiplexed using bcl2fastq (version 2.20.0.422) to convert BCL files to FASTQ files. A reference for GRCh38 was created following the Cell Ranger (version 6.0.1) protocol. Gene expression matrices for downstream analyses were calculated using the ‘count’ function of Cell Ranger and default parameters.

*Preprocessing of snRandom‐Seq Data*: First, the primer sequences and extra nucleotides generated in the dA‐tailing step were trimmed from the raw sequencing data. Then, for each Read1, we extracted the UMI (8 nt) and cell‐specific barcode (30 nt) and merged the sorted barcodes, which were uniquely assigned to the same acceptor barcode with a Hamming distance of 2 nt or less. Read2 was generated into a gene expression matrix by the STARsolo module in STAR (2.7.10a) with reasonable parameters. To determine the number of nuclei in each sample, we plotted a scatterplot of log10(genes) for each possible barcode and used the position of the minimum of the maximum log10(genes) value as the threshold: only barcodes with gene counts above this threshold were used for downstream analysis.

*Clustering*: The gene expression matrix generated from barcode filtering excluded mitochondrial RNA and ribosomal RNA. Subsequently, single‐nucleus RNA sequencing (snRNA‐seq) data analysis and visualization were performed using the Seurat v4 toolkit. The workflow included preprocessing, integration, visualization, clustering, cell type identification, and differential expression detection. Nuclei with fewer than 200 genes are filtered out, as well as genes detected in less than 3 nuclei. To integrate the snRNA‐seq dataset, the sctransform function in Seurat was employed for count normalization, and canonical correlation analysis (CCA) was used for integration. For each sample, 4000 anchors were determined, and the snRNA‐seq dataset was integrated with the IntegrateData function using 50 dimensions. The integrated dataset was constructed as a shared nearest neighbour (SNN) graph through principal component analysis (PCA), FindNeighbours with 50 PCs, and FindClusters with a resolution of 0.5. Clustering was visualized using uniform manifold approximation and projection (UMAP), which was implemented in Seurat. The main components in Seurat were used to manually determine the cell type identity of each cluster based on a published list of marker genes. The FindAllMarkers function in Seurat was utilized to identify the marker genes, and filtering conditions (only.pos = TRUE, min.pct = 0.25, logfc.threshold = 0.25) were applied to ensure consistency. The stress signature scores were calculated using AddModuleScore, with genes derived from Van et al., 2017 and Denisenko et al., 2020.^[^ ^37^, ^39^ ^]^

*CNV Analysis and Identification of Tumor Cells*: As there was no corresponding adjacent normal tissue among our samples, we utilized CopyKAT (Copy Number Variation and karyotype analysis in Tumors^[^ ^40^ ^]^) software to distinguish malignant cells from normal cells by calculating the copy number variation (CNV) level for each cell. This software clustered the processed UMI data and first selected cells with high confidence in diploidy. Then, hierarchical clustering was used to identify tumor cells with significant differences from normal cells. Nonsignificant genomes were identified one by one using a Gaussian mixture model (GMM). Ultimately, gene expression profiles of malignant and normal cells were obtained. In addition to the automated identification of malignant cells by CopyKAT, we calculated a CNV score to aid in identifying malignant cells. The CNV score of each cell was calculated as the quadratic sum of CNV regions.^[^ ^41^ ^]^

*Pseudotime Analysis*: In this study, we utilized the Monocle (v.2.8.0) package to perform a comprehensive analysis of cell differentiation and fate determination.^[^ ^42^ ^]^ To explore related clusters, we extracted the subset of raw data with cluster information and used the ‘dispersionTable’ function to calculate the variance in each gene's expression across cells. Variable genes were selected based on the mean expression level, and the data's dimensionality was reduced to two components using the ‘max_components = 2, method = DDRTree’ parameter. We used the ‘orderCells’ function to order the cells in pseudotime, and the resulting trajectory was visualized using the ‘plot_cell_trajectory’ function in Monocle. To specify the beginning of the trajectory, we ran ‘orderCells’ again and set the ‘root_state’ argument. We then selected the branch point to analyze branches in differentiation trajectories and used BEAM to analyze pseudotime‐dependent or branch‐dependent genes. The ‘plot_genes_branched_heatmap’ function was used to visualize genes that were significantly branch dependent.

*Identification of lncRNA‐mRNA Pairs*: To accomplish the identification of lncRNA‐mRNA pairs, we employed the LncPairs algorithm.^[^ ^24^ ^]^ The detailed analysis steps are as follows: 1) A gene×cluster expression matrix is constructed, using the top 2000 highest varying gene‐based single‐cell expression matrix. Each gene expression is averaged based on clusters. 2) The gene×cluster is then divided into two matrices, namely mRNAcluster and lncRNAcluster. 3) The correlation between the two matrices – mRNAcluster and lncRNAcluster – is calculated. Further emphasis is placed on lncRNA‐mRNA pairs exhibiting a Pearson Correlation Coefficient (PCC) that exceeds 0.85. 4) Using the residual lncRNA‐mRNA pairs, the paircluster matrix is then built. 5) The Cosine similarity approach serves to distinguish the cluster‐specific lncRNA‐mRNA pairs. 6) Lastly, any pairs with a similarity score below 0.95 are eliminated from the data set.

*Enrichment Analysis*: All genes that were found to be differentially expressed, including those enriched in specific clusters, were subjected to pathway enrichment analysis using clusterProfiler.^[^ ^43^ ^]^ The pathways that showed high statistical significance were annotated to specific biological processes.

### In Situ Hybridization

Selection of the corresponding FFPE wax sections for validation purposes. The labeled DNA probes were hybridized with the samples using the FISH in situ hybridization kit (Catalog No: C007). After completion of the hybridization reaction, cell nuclei were stained with DAPI. Fluorescence microscopy was used to observe the cells at a wavelength of 340 nm, and the hybridization signals were observed at a wavelength of 552 nm.

## Conflict of Interest

The authors declare no conflict of interest.

## Author Contributions

W.J. and X.Z. contributed equally to this work. Conceptualization: W.J., L.F., and H.C. Data Curation: X.Z., Q.C., X.L., and H.C. Experimental Operations: W.J., Z.X., Y.Z., L.D., F.L., and W.Z. Supervision: L.Z., Y.W., L.F., and H.C. Writing—original draft: W.J., X.Z., and H.C. Writing—review & editing: L.F. and H.C.

## Supporting information



Supporting Information

Supporting Information

## Data Availability

The snRandom‐seq scRNA datasets obtained in this study have been deposited in National Genomics Data Center under accession number PRJCA019001 for all FFPE puncture samples.
