# Supplementary material for: High‐Throughput Single‐Nucleus RNA Profiling of Minimal Puncture FFPE Samples Reveals Spatiotemporal Heterogeneity of Cancer
Source: Adv Sci (Weinh). 2024 Dec 4;12(4):2410713. doi: 10.1002/advs.202410713 (PMC11789576; doi:10.1002/advs.202410713)
Supplement: Supplementary file 1 — Supporting Information [file ADVS-12-2410713-s002.docx]

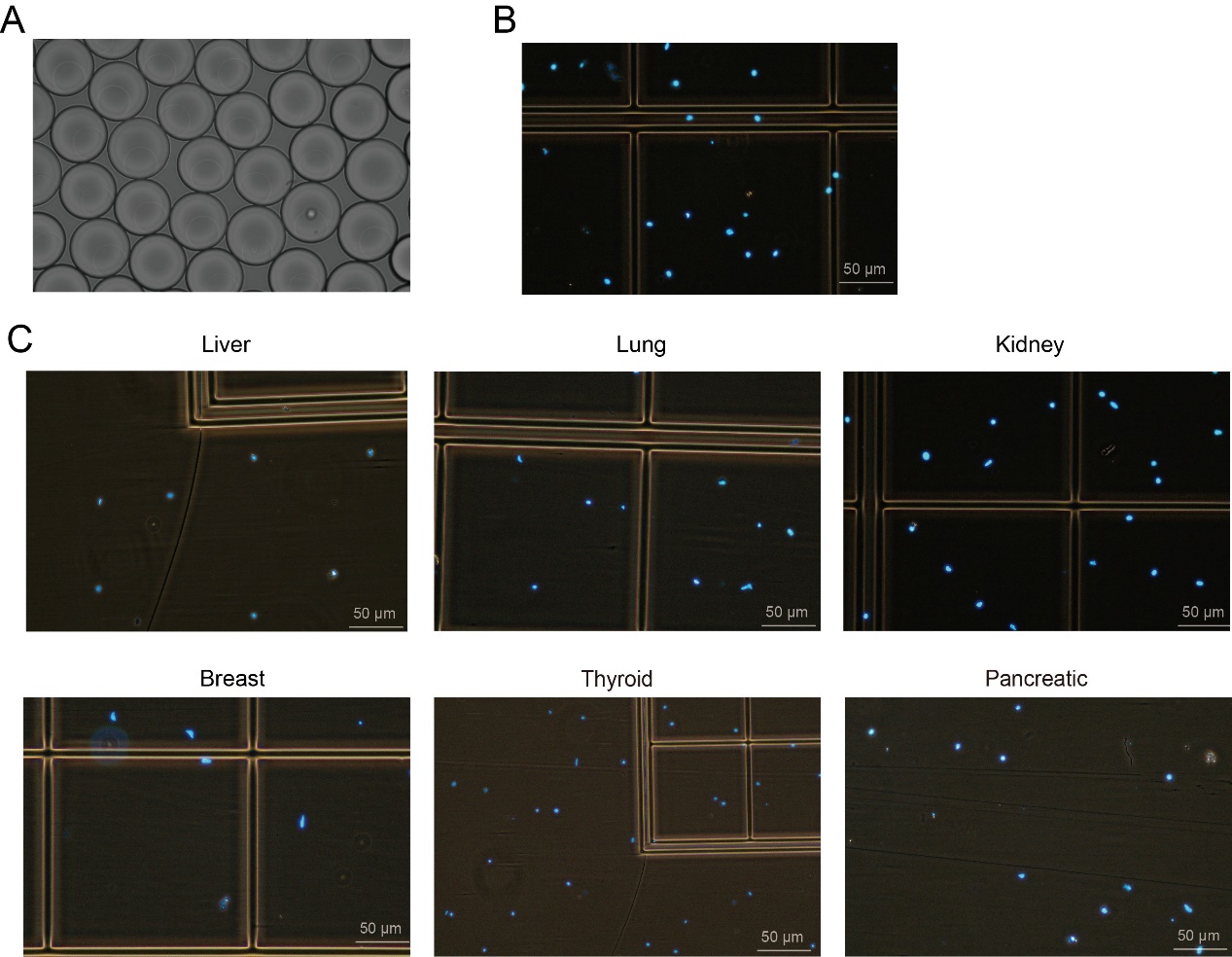


**Figure S1 Microscopic view reveals cell encapsulation and cancer nuclei details.** (A) image of a droplet that has been encapsulated with a single bead, a single nucleus, and a mixture of reagents. (B-C) DAPI-stained image of a single nucleus extracted from different cancer tissues. This includes samples of colorectal cancer liver metastasis, liver cancer, lung cancer, kidney cancer, breast cancer, pancreatic cancer, and thyroid cancer.


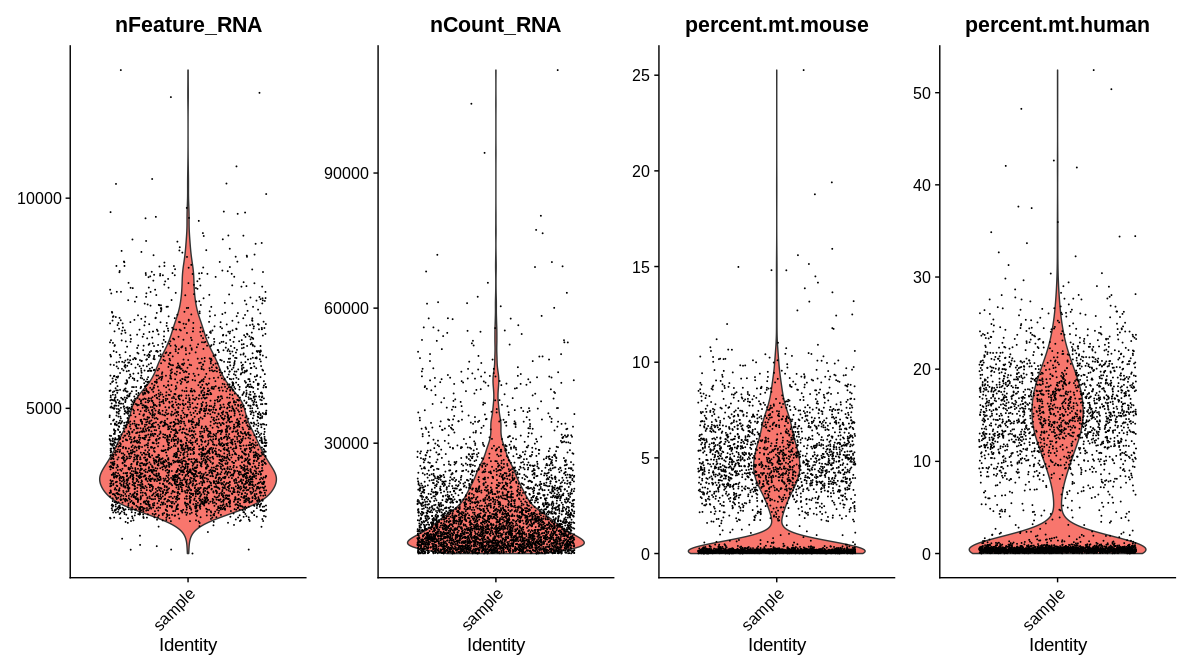


**Figure S2** The violin plot presents the data of a nuclei mixture sample comprising 293T (human) and 3T3 (mouse) cells, including the number of features detected per cell (which can be understood as genes), the number of reads detected per cell, and the proportion of human and mouse mitochondrial genes in each cell.


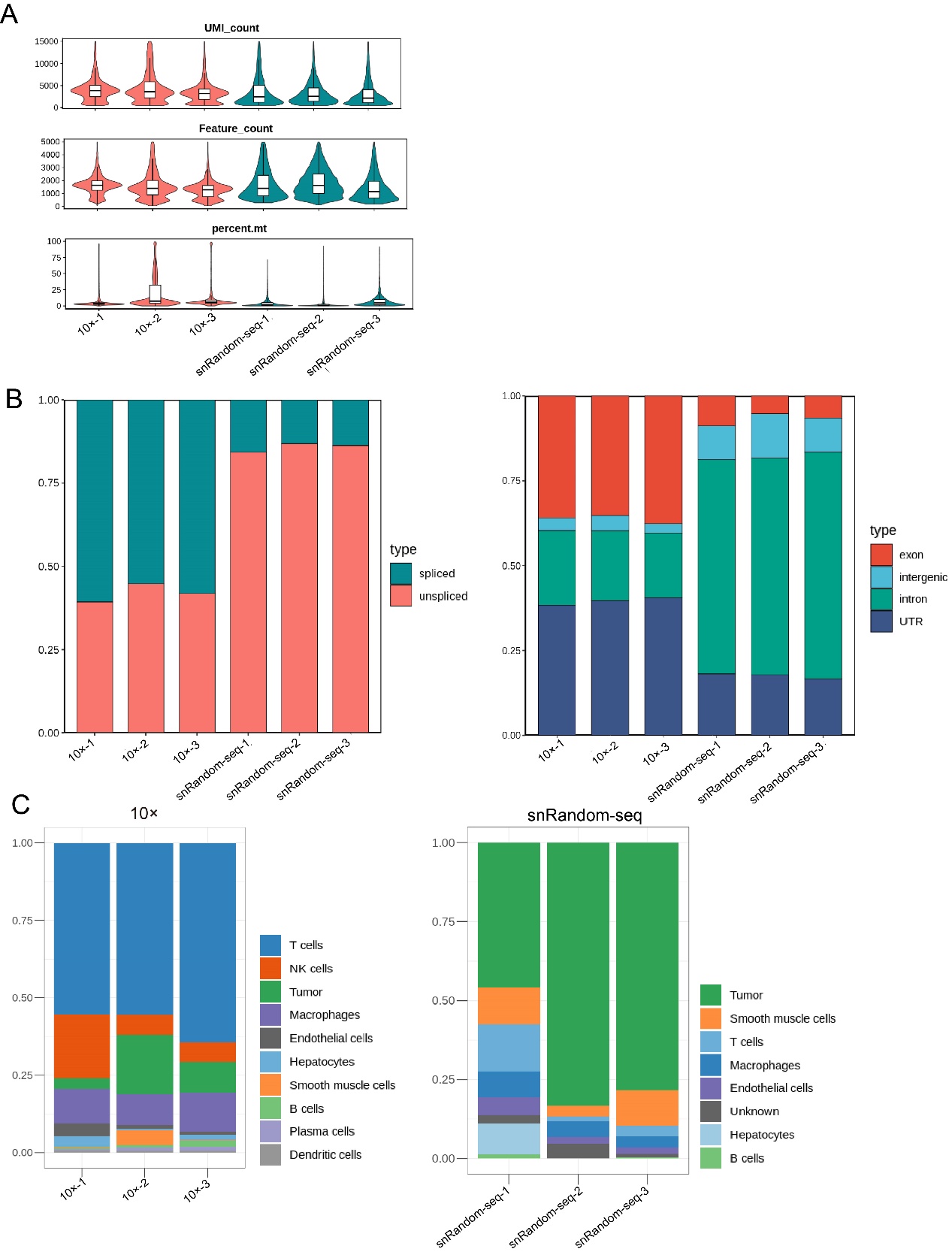


**Figure S3 Demonstration of data characteristics.** (A) Violin plots displaying the counts, detected features, and mitochondrial proportions for each sample. (B) The proportion of spliced and unspliced reads in each sample data (left). The comparison of sequencing data mapping situation, which refers to the proportion of mapping to intronic regions, exonic regions, intergenic regions, and UTR regions (right). (C) Distribution of cell type proportions between fresh biopsy samples and paraffin-embedded biopsy samples.


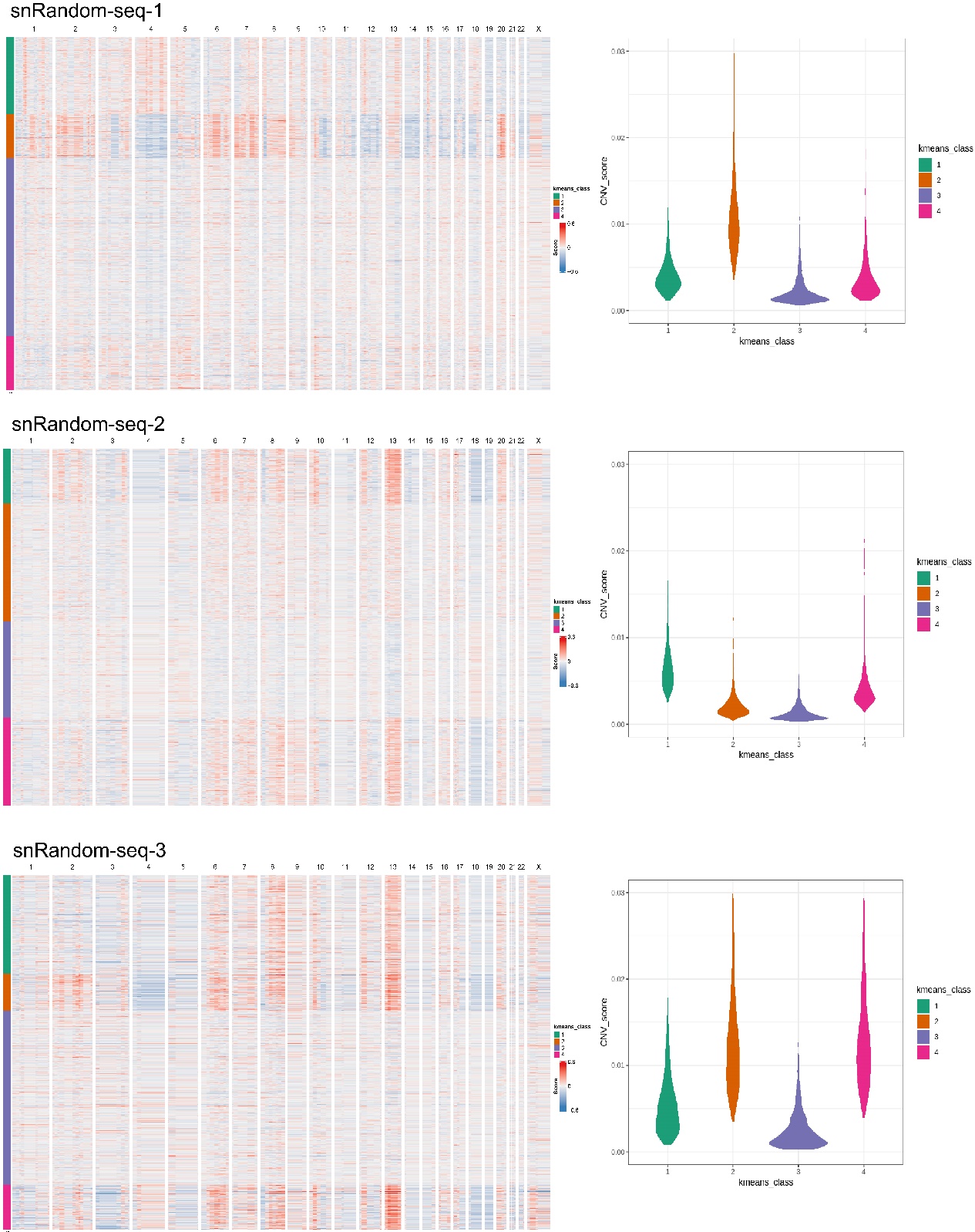


**Figure S4** The CNV analysis results of single-cell transcriptome data under optimized snRandom-seq technology are shown in the left panel of the figure, displaying the CNV variations on chromosomes 1-22 and X. Red represents Gain, blue represents loss. The different bars on the left side of the heatmap represent clustering results based on the CNV results, with different colors representing different clusters. On the right side, CNV scores are assigned to different clusters to further determine whether they are tumor cells, with higher scores indicating tumor cells.


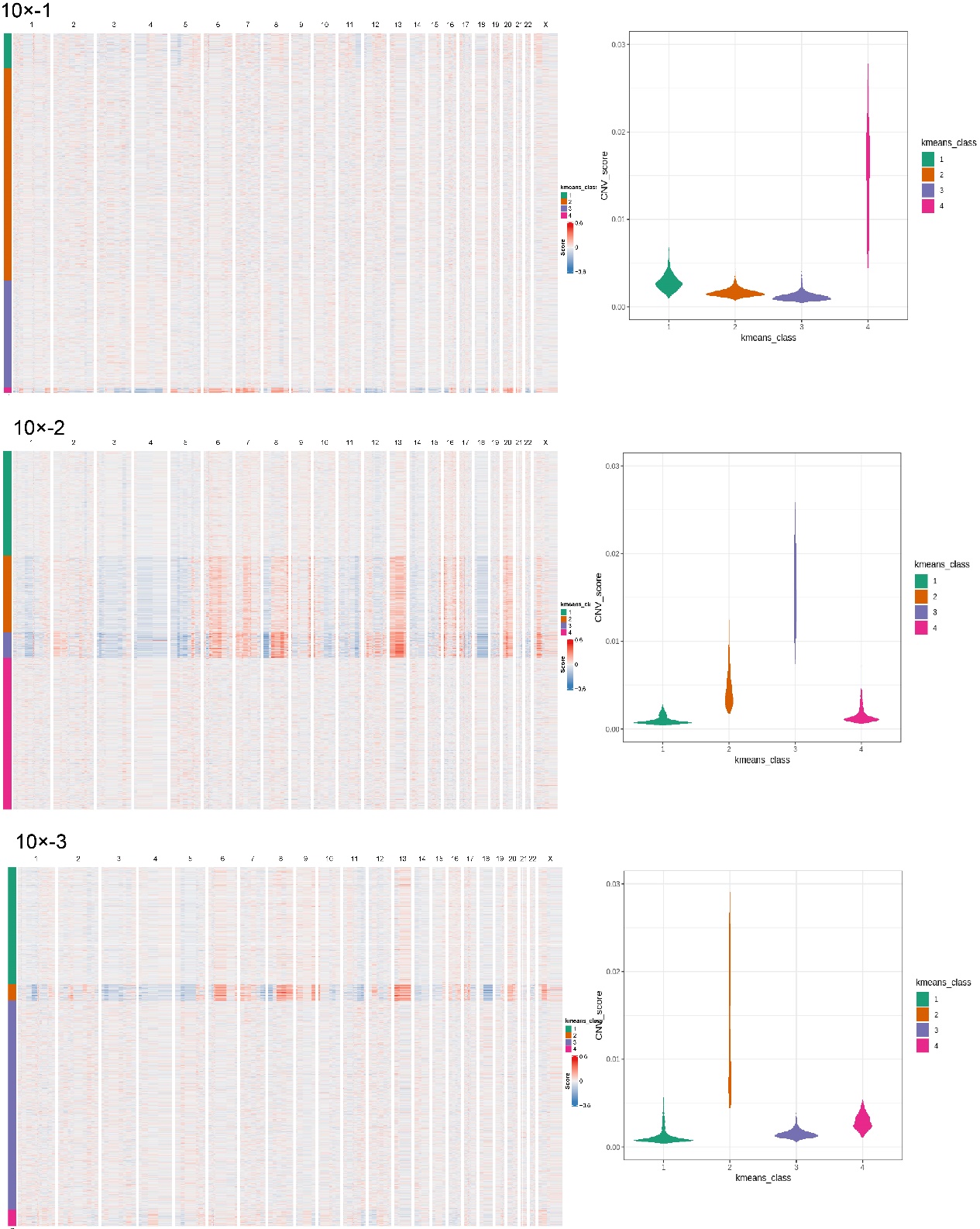


**Figure S5** The CNV analysis results of single-cell transcriptome data under 10× Genomics technology are shown in the left panel of Figure, displaying the CNV variations on chromosomes 1-22 and X. Red represents Gain, blue represents loss. The different bars on the left side of the heatmap represent clustering results based on the CNV results, with different colors representing different clusters. On the right side, CNV scores are assigned to different clusters to further determine whether they are tumor cells, with higher scores indicating tumor cells.


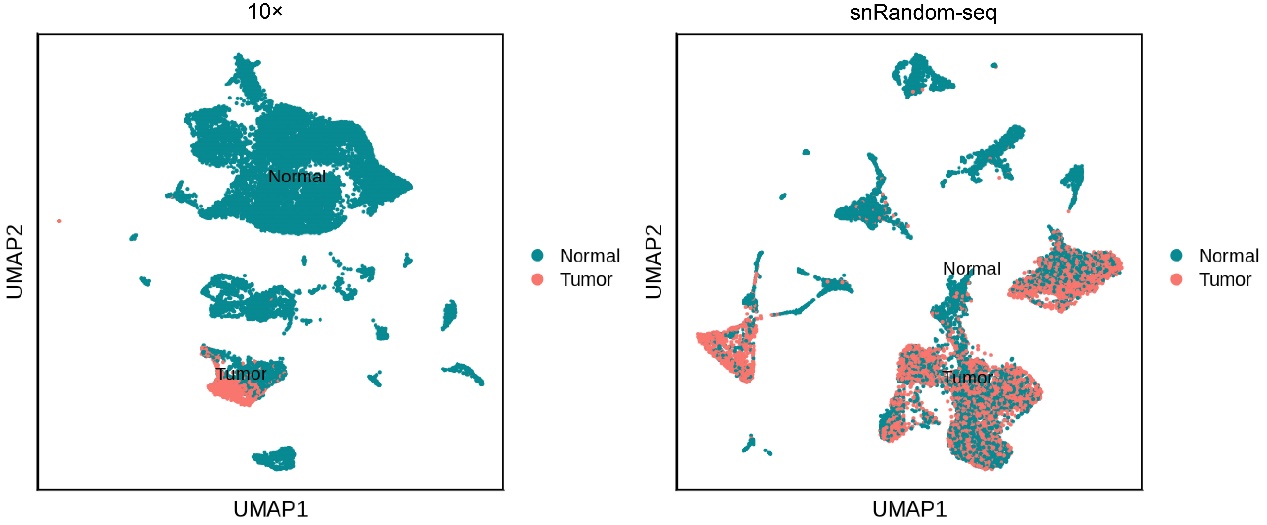


**Figure S6** The positions of tumor cells identified by CNV analysis in samples (The left side consists of 10× samples, while the right side consists of optimized snRandom-seq samples) are shown in UMAP plot. Pink indicates tumor cells, while dark blue represents normal cells.


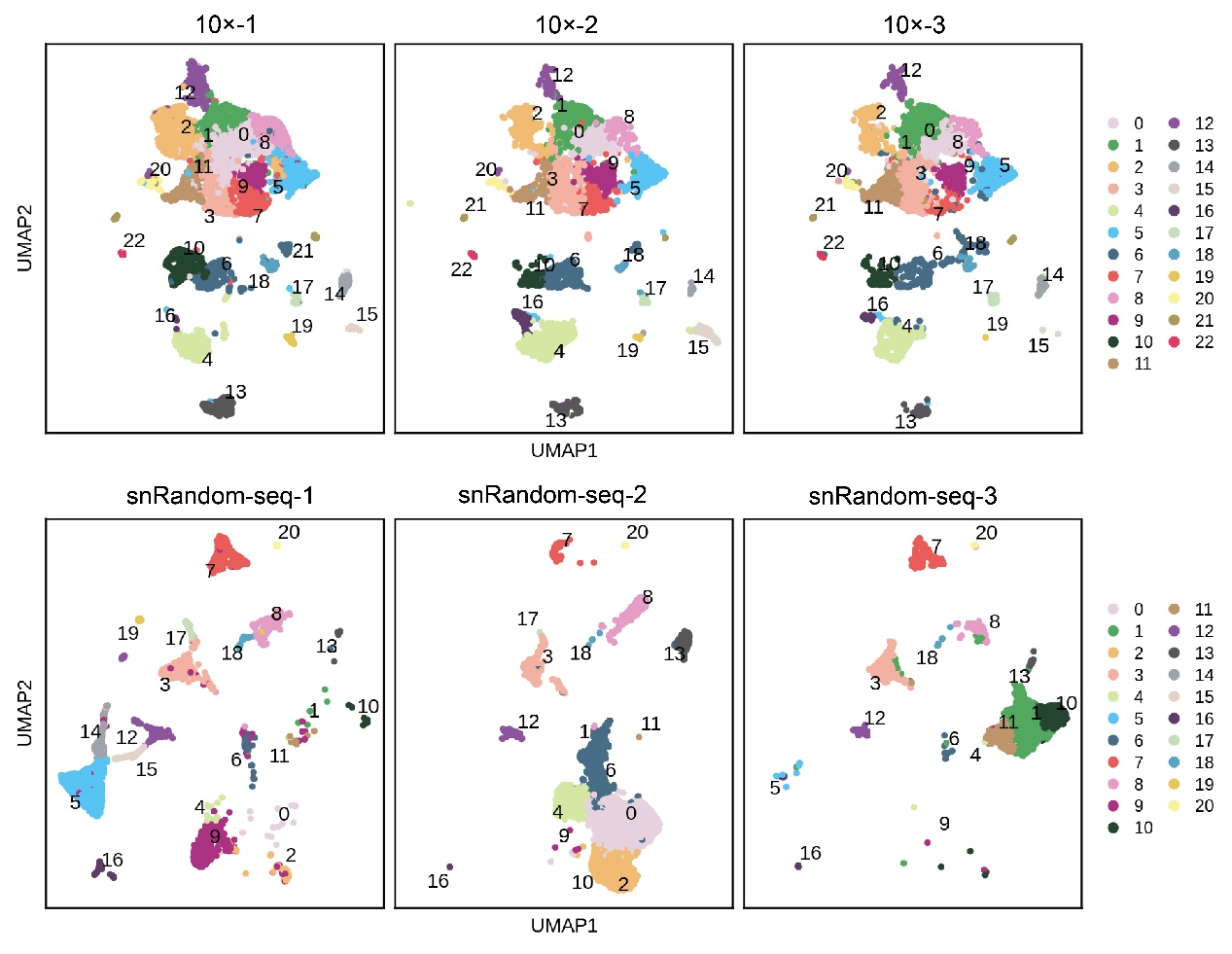


**Figure S7** Figure showing the integration of pairwise data, The upper panel shows the results from fresh samples processed by 10× genomics, while the lower panel displays the results from FFPE samples processed by optimized snRandom-seq.


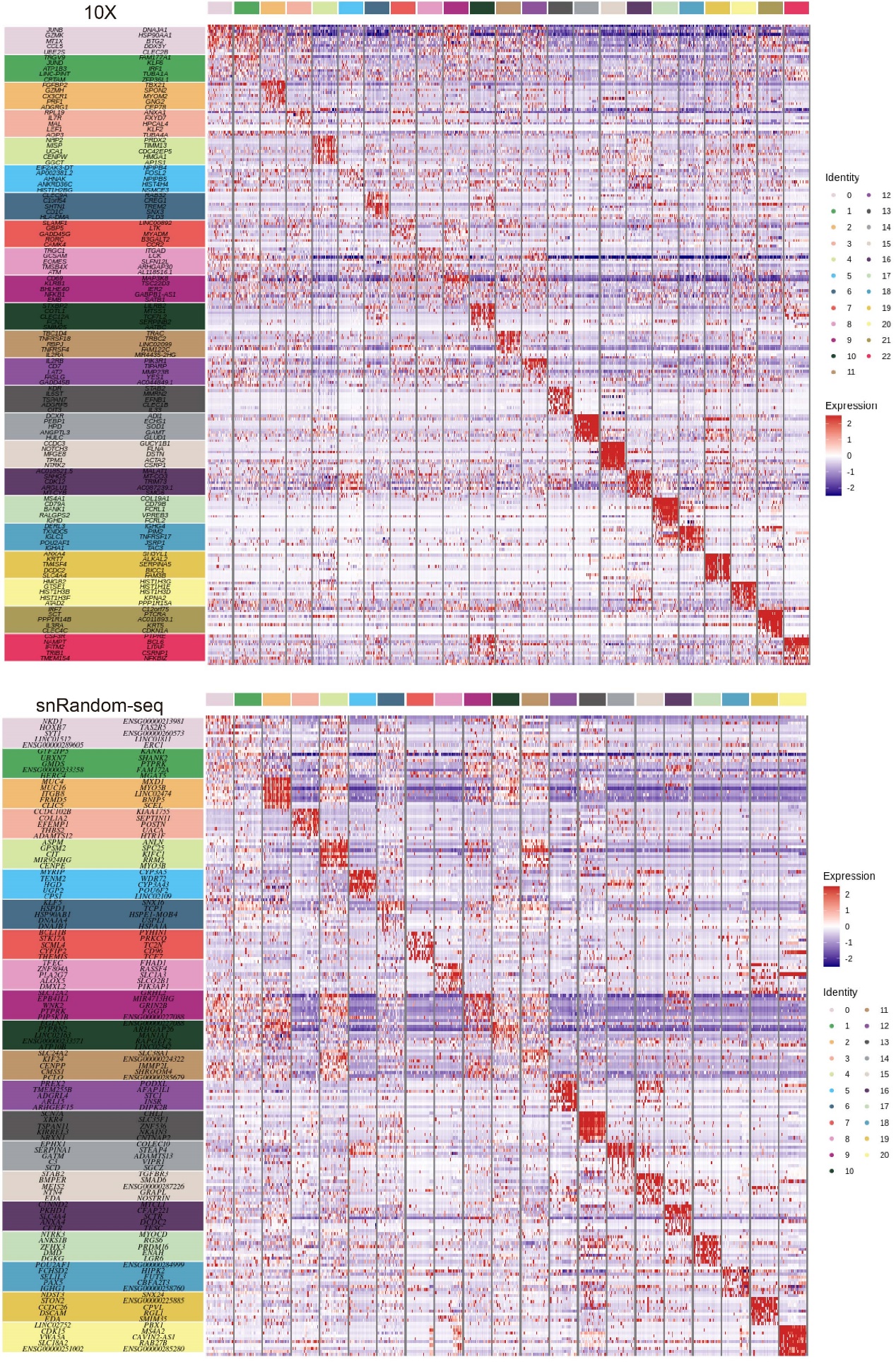


**Figure S8** The heatmap shows the top 10 differentially expressed genes between different cell clusters, with the gene names shown on the coordinates.


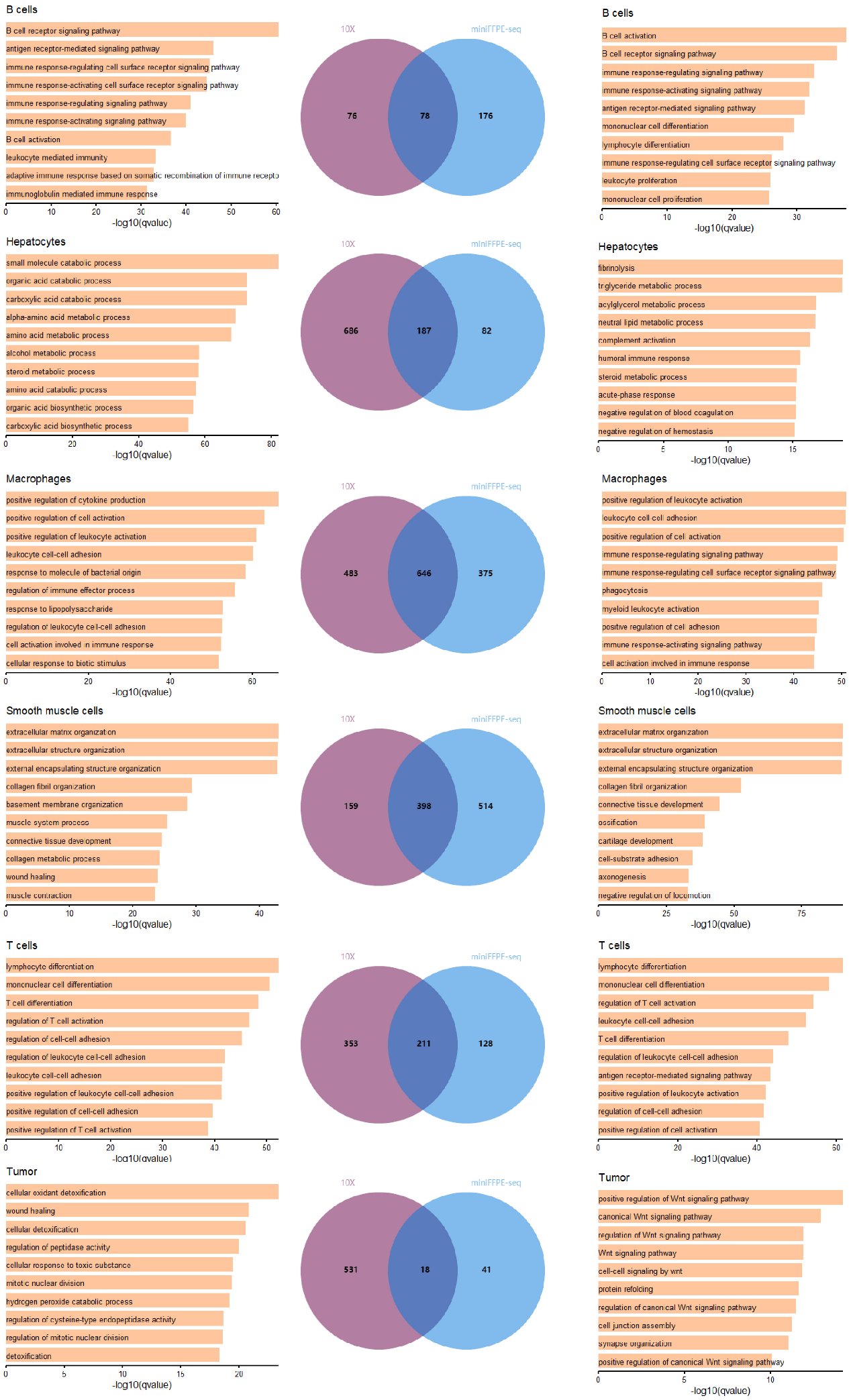


**Figure S9** The results of GO enrichment analysis for differentially expressed genes (DEGs) identified in cell type identification under two techniques are presented in Figure S4. The bar graphs display the top 10 enriched pathways for each cell type, with the leftmost representing the 10× Genomics technique, the rightmost representing the optimized snRandom-seq technique, and the middle section illustrating a Venn diagram showing the intersection of enrichment results between the two methods.


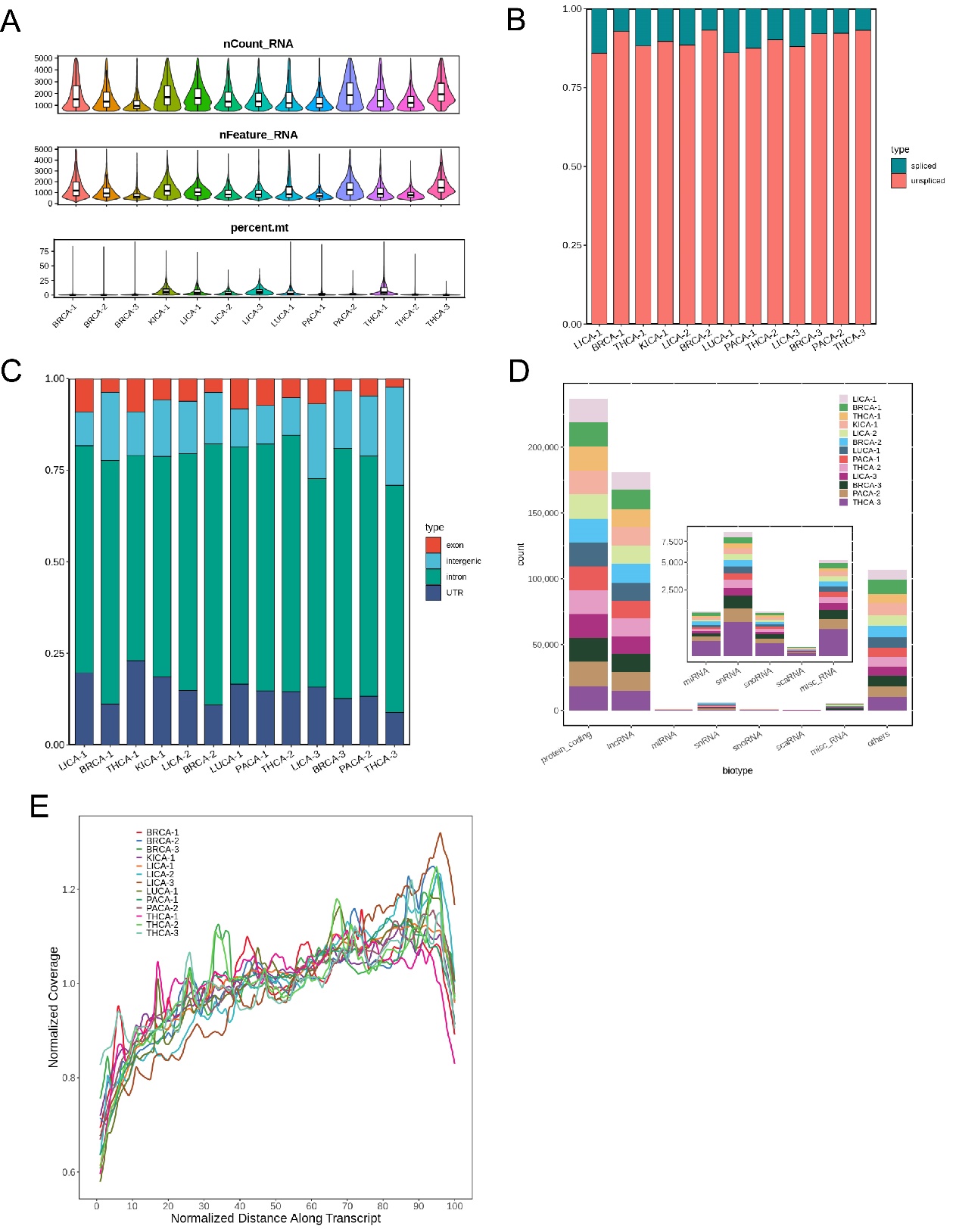


**Figure S10** Data analysis of 6 types of cancer across 13 samples using optimized snRandom-seq technology. (A) Violin plots displaying the counts, detected features, and mitochondrial proportions for each sample. (B) Presentation of the proportion of spliced and unspliced reads in each sample. (C) Stacked bar chart illustrating the types and corresponding numbers of genes involved in the data for each sample, with each color representing one sample. (D) Coverage of sample data across different genomic regions (exons, introns, intergenic regions, UTRs). (E) Coverage of different transcript regions across various samples.


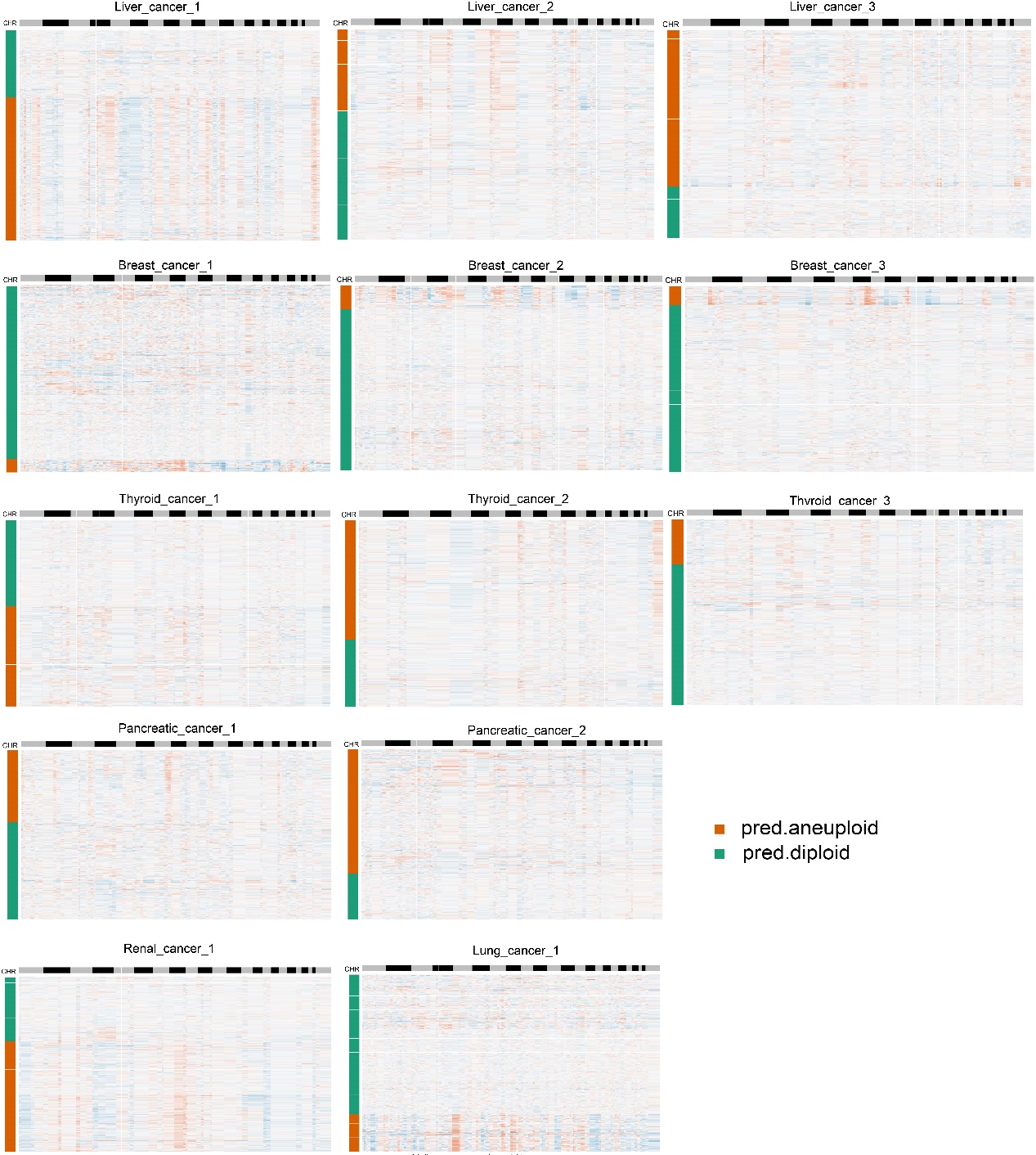


**Figure S11** A heatmap presentation of CNV analysis results for 13 single-cell transcriptome data samples from 6 types of cancer using the optimized snRandom-seq technique is shown, displaying CNV variations on chromosomes 1-22 and X. This includes samples of liver cancer, lung cancer, kidney cancer, breast cancer, pancreatic cancer, and thyroid cancer. The orange color represents gains, while the green color represents losses. The different bars on the left side of the heatmap indicate tumor cell identification results based on CNV analysis, with the orange bar representing non-polyploidy and the green bar representing polyploidy.


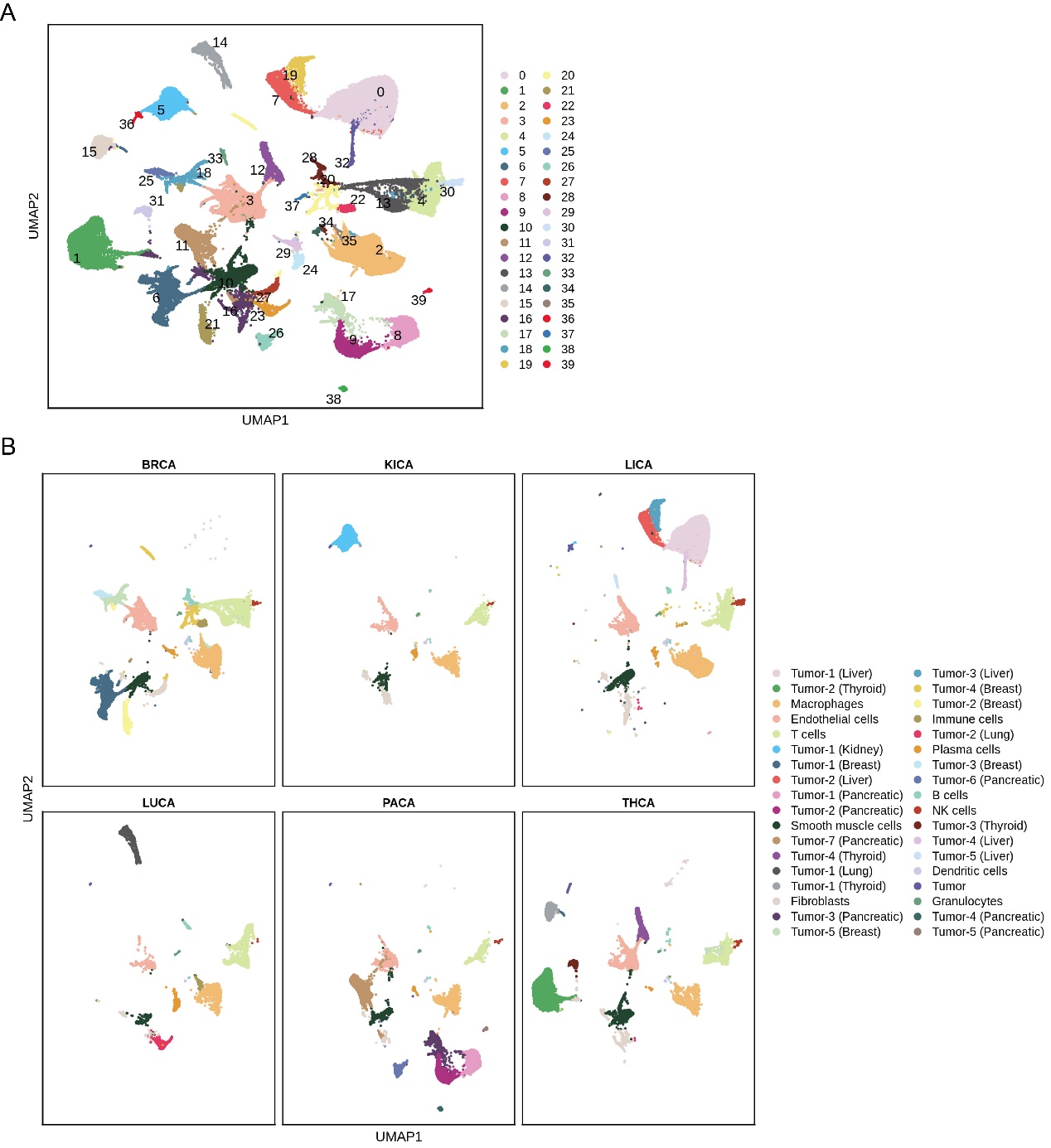


**Figure S12** UMAP was used to display the integrated clustering results of 13 samples from six different types of cancer (include liver cancer, lung cancer, kidney cancer, breast cancer, pancreatic cancer, and thyroid cancer). The following figures present the clustering results of each cancer type after integration.


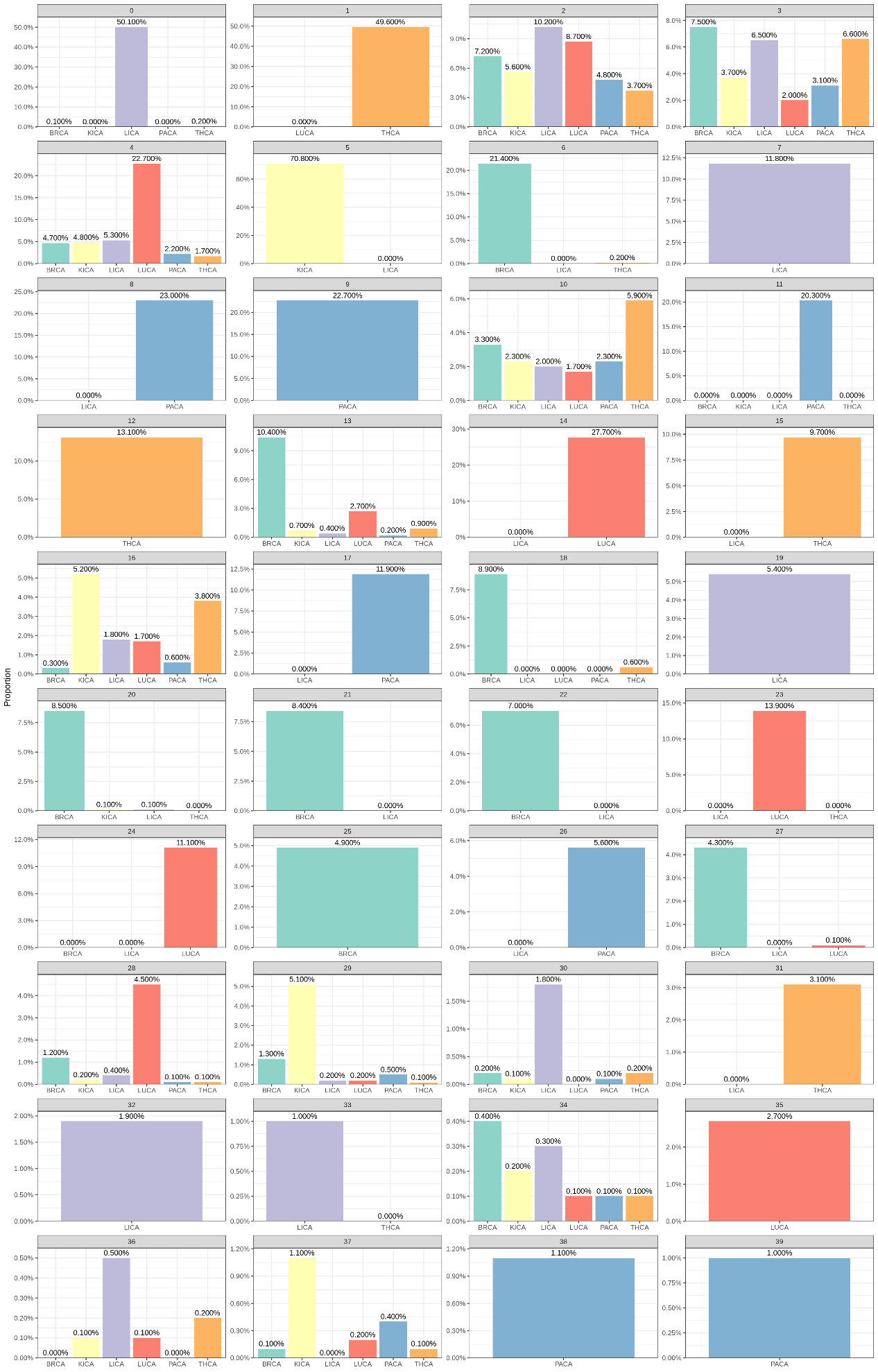


**Figure S13** A bar chart displaying the proportions of six cancer types across various cell clusters


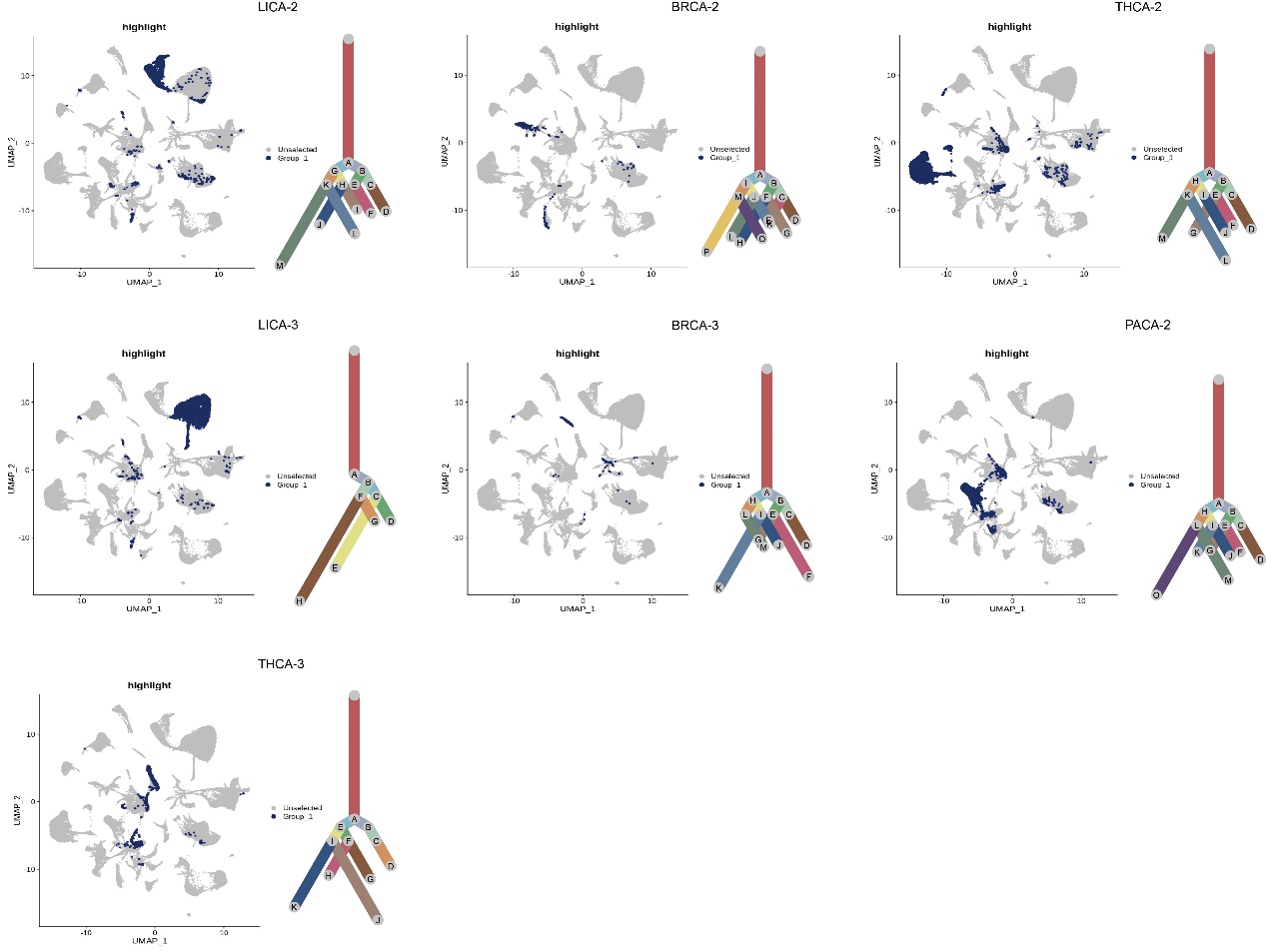


**Figure S14** The UMAP heatmap displays the positional information of the identified tumor cells in the integrated UMAP plot for the remaining samples, with the corresponding evolutionary tree information of the tumor cells shown on the right.


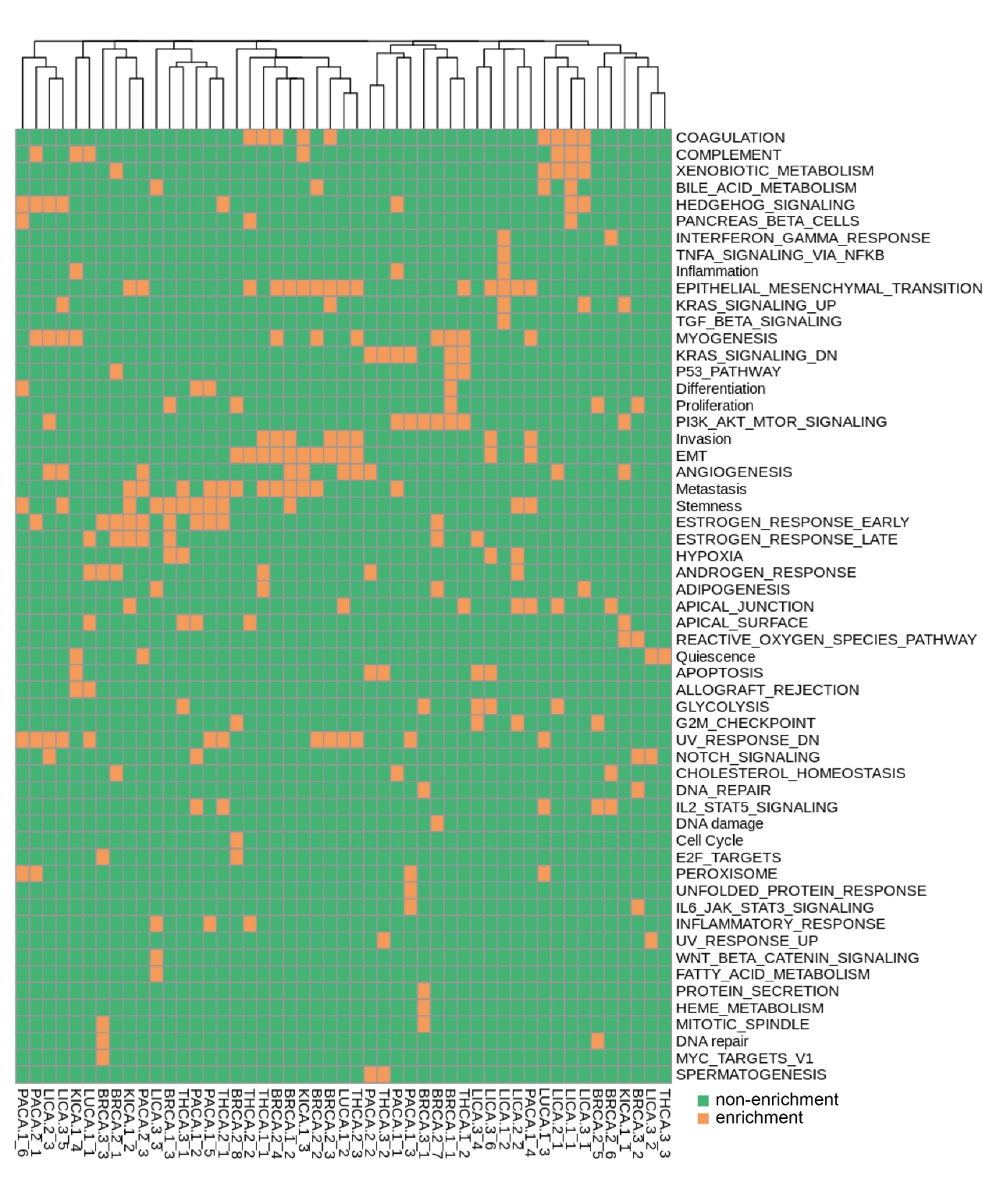


**Figure S15** MSigDB gene set enrichment analysis of top 50 genes for each program using MSigDB curated H gene set collection. DE = differential expression; MSigDB = Molecular Signatures Database.


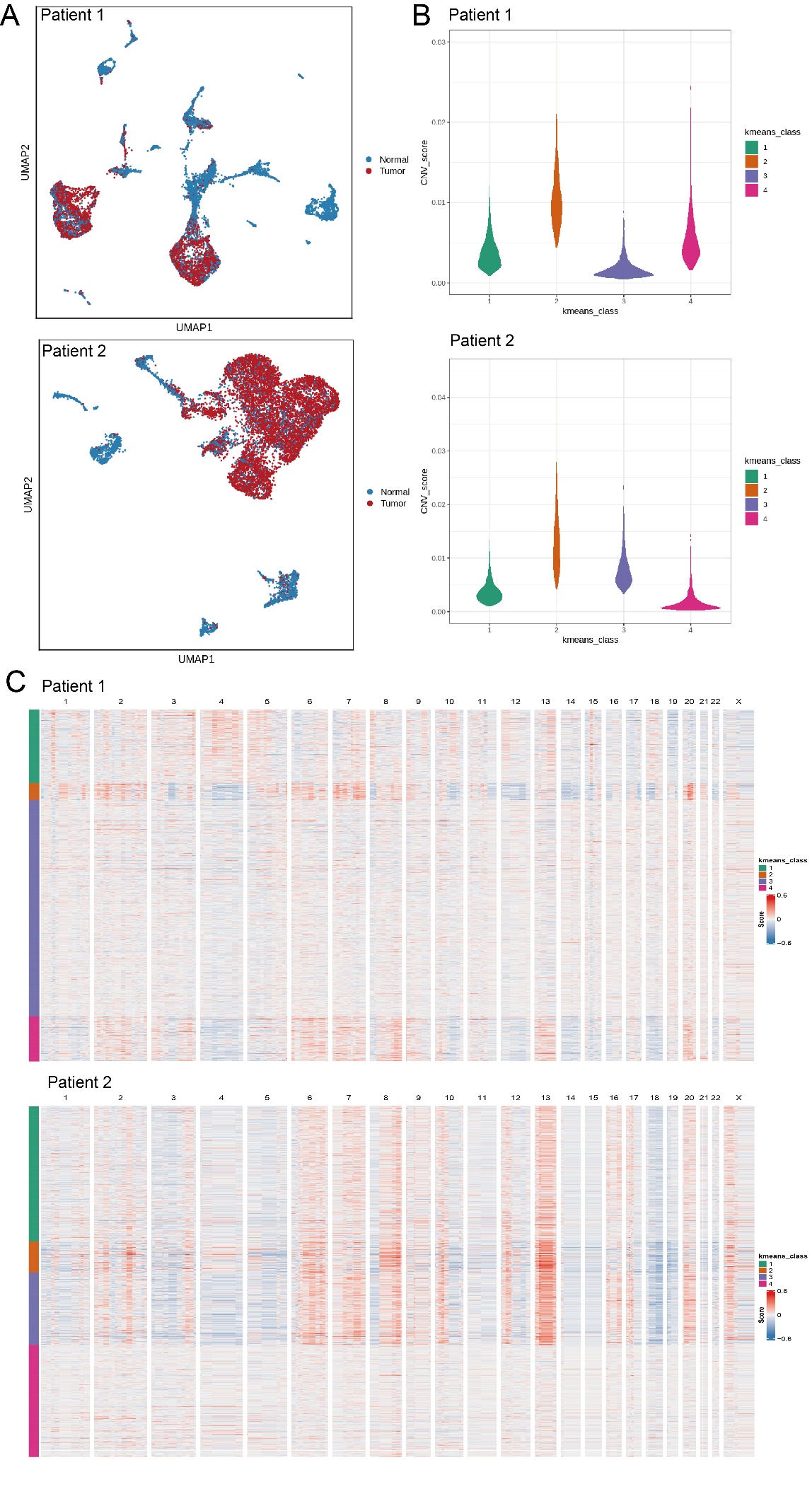


**Figure S16** Results of copy number variations analysis in patient's tumor cells. (A) The positions of tumor cells identified by CNV analysis in two patient samples are shown in UMAP plot. (B) Violin plots display the distribution of CNV scores in different clusters of the two patient samples, indicating whether they are tumor cells. A higher score indicates a higher likelihood of being a tumor cell. (C) Heatmaps display the CNV variations on chromosomes 1-22 and X in the two patient samples. Red represents gain, blue represents loss. The bars on the left side of the heatmap indicate clustering results based on the CNV results, with different colors representing different clusters. CNV scores are assigned to different clusters on the right side to further determine whether they are tumor cells, with higher scores indicating a higher likelihood of being tumor cells.


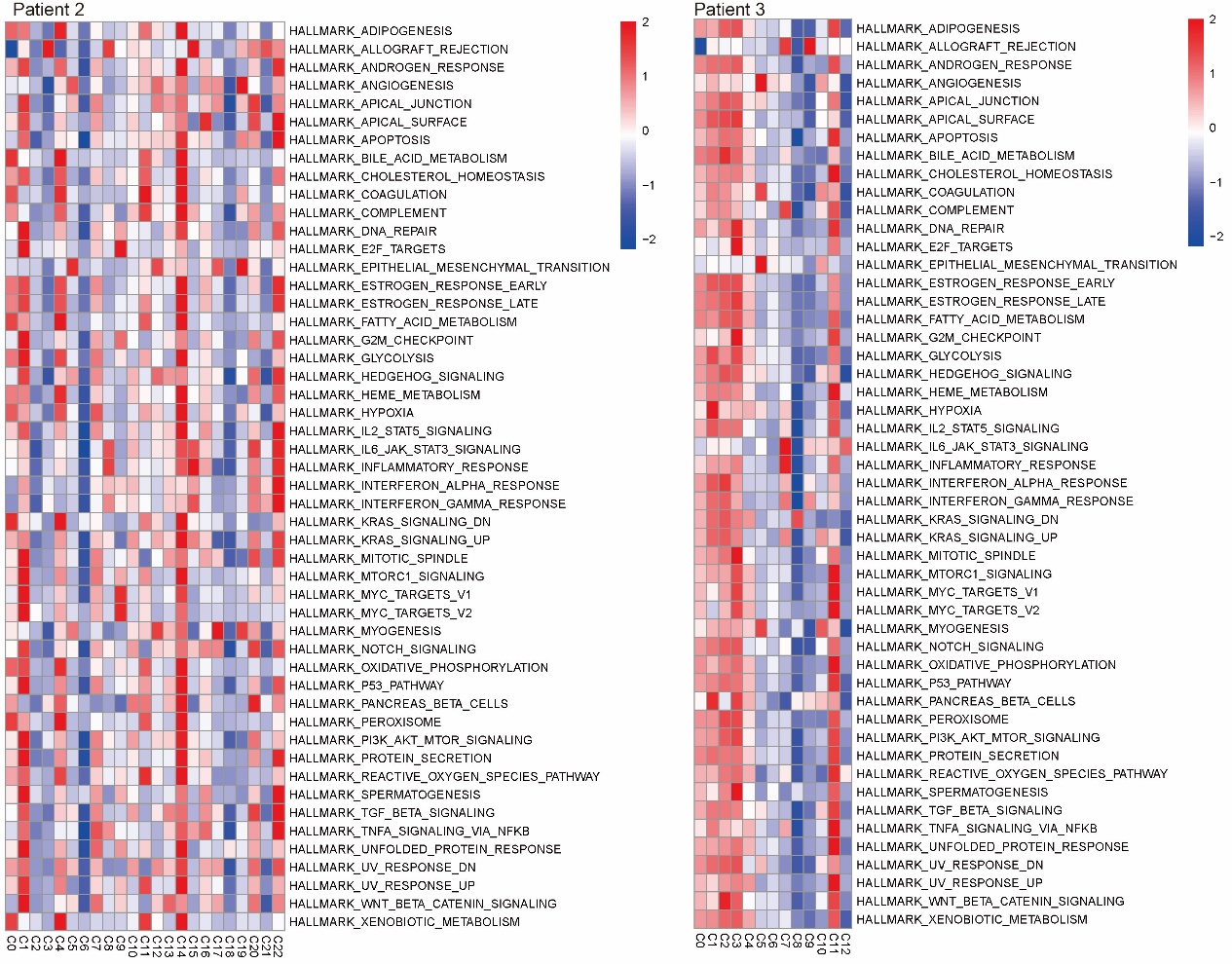


**Figure S17** MSigDB gene set enrichment analysis of cluster DE genes using MSigDB curated (H) gene set collection. C0 = Cluster #0; DE = differential expression; MSigDB = Molecular Signatures Database.


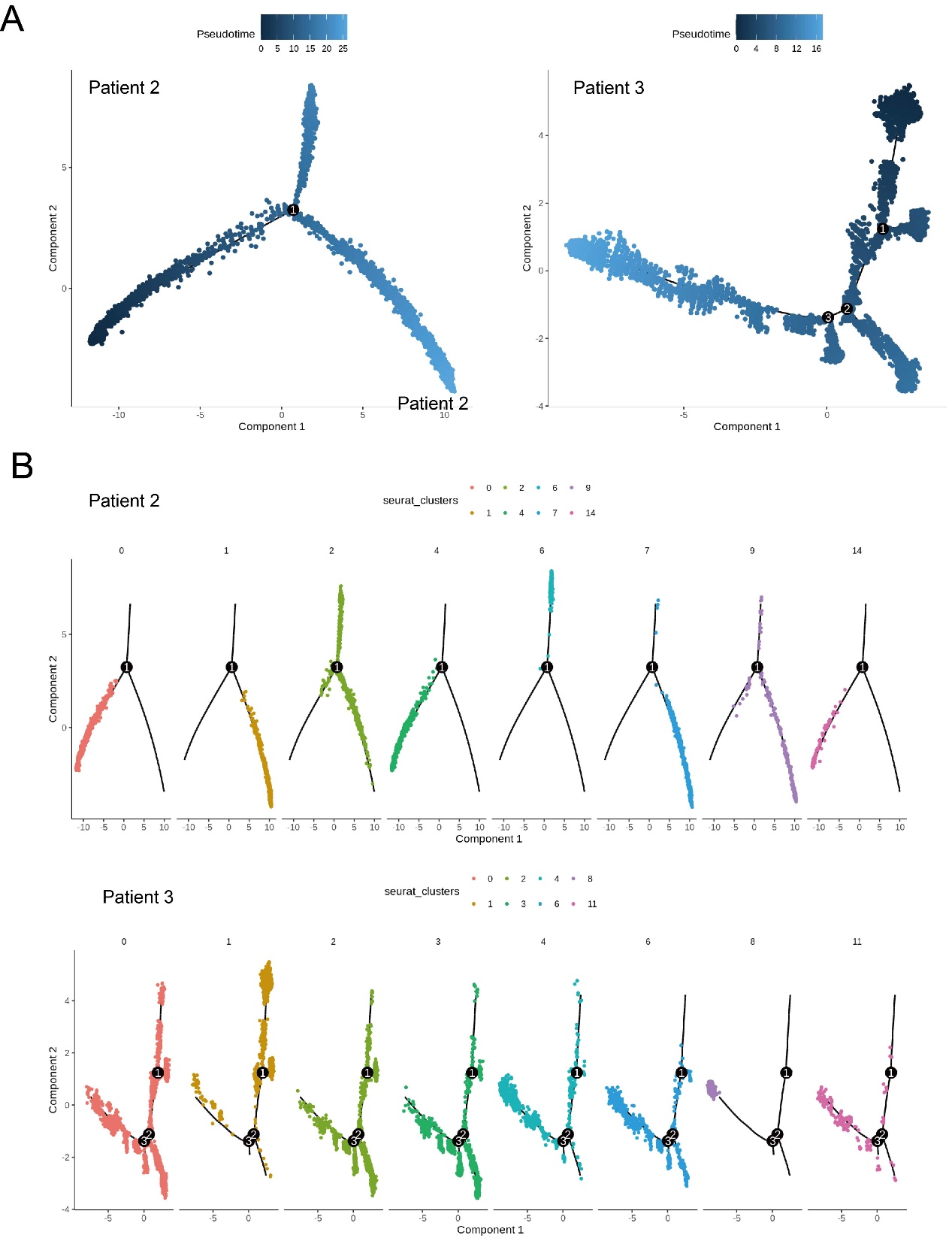


**Figure S18** Results of developmental trajectory analysis in patient's tumor cells. (A) displays the developmental trajectories of tumor cells from two patients, where the color gradient from dark to light represents the direction of the trajectory. (B) provides the positional information of different tumor cell clusters along the developmental trajectory.


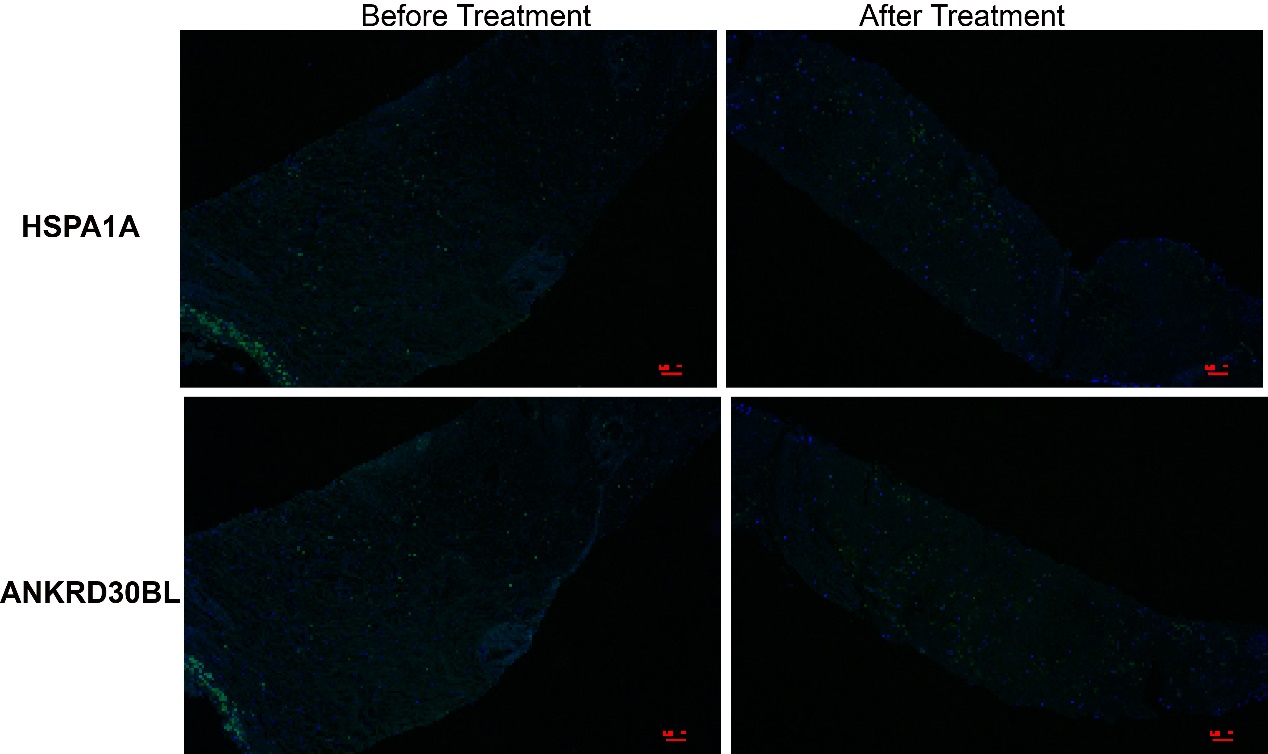


**Figure S19** The FISH experimental results showing the unique cell cluster #4 in Patient 2 post-treatment exhibit two marker genes (HSPA1A and ANKRD30BL). The green signals indicate the expression of the respective genes.


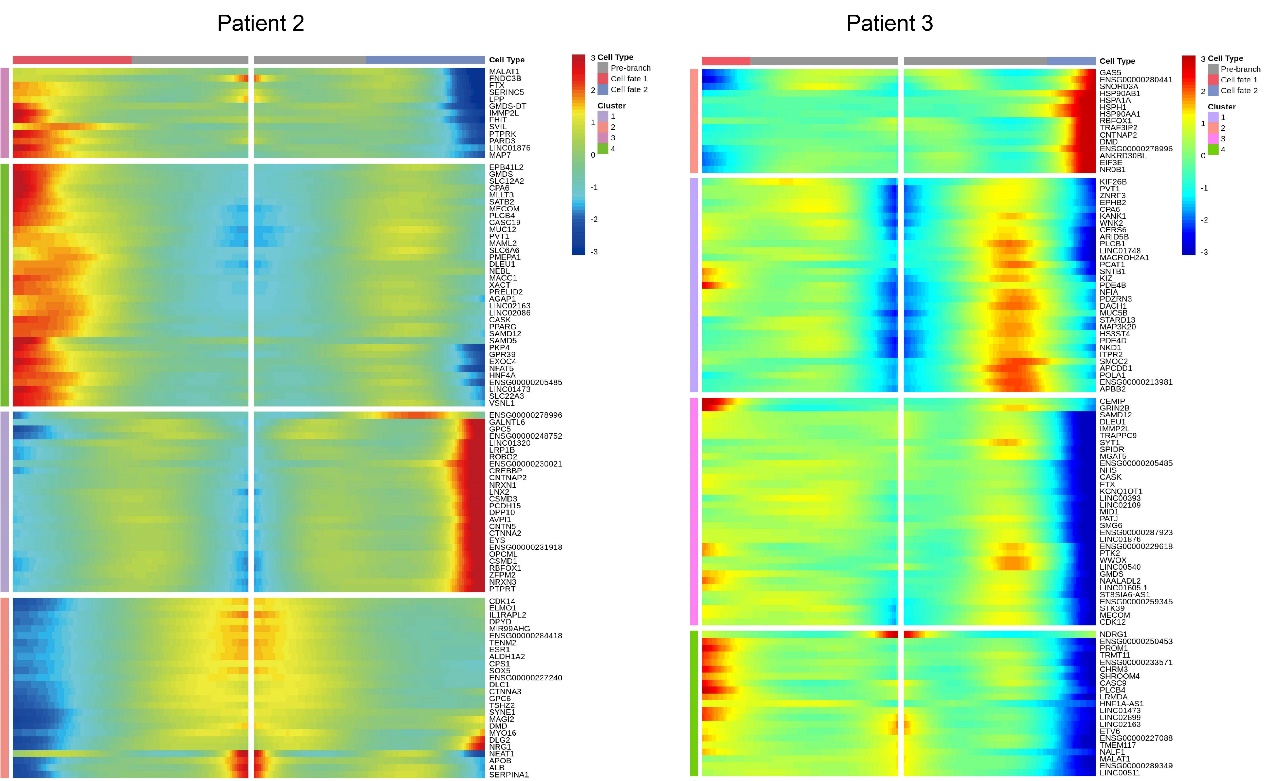


**Figure S20** The heatmap displays the expression changes of genes at the branching points of the developmental trajectories, with the top 100 genes showing the most significant differences selected.
